# Intelligent Molybdenum Disulfide Complexes as a Platform for Cooperative Imaging‐Guided Tri‐Mode Chemo‐Photothermo‐Immunotherapy

**DOI:** 10.1002/advs.202100165

**Published:** 2021-06-18

**Authors:** Wei Hu, Tingting Xiao, Du Li, Yu Fan, Lingxi Xing, Xipeng Wang, Yulin Li, Xiangyang Shi, Mingwu Shen

**Affiliations:** ^1^ State Key Laboratory for Modification of Chemical Fibers and Polymer Materials College of Chemistry, Chemical Engineering and Biotechnology Donghua University Shanghai 201620 P. R. China; ^2^ Department of Gynecology and Obstetrics XinHua Hospital Affiliated to Shanghai Jiao Tong University School of Medicine Shanghai 200092 P. R. China; ^3^ The Key Laboratory for Ultrafine Materials of Ministry of Education State Key Laboratory of Bioreactor Engineering Engineering Research Center for Biomedical Materials of Ministry of Education School of Materials Science and Engineering East China University of Science and Technology Shanghai 200237 P. R. China; ^4^ CQM‐Centro de Quimica da Madeira Universidade da Madeira Funchal 9020‐105 Portugal

**Keywords:** immunogenic cell death, molybdenum disulfide, multimode imaging, polydopamine, tri‐mode chemophotothermo‐immunotherapy of tumors

## Abstract

Design of new nanoplatforms that integrates multiple imaging and therapeutic components for precision cancer nanomedicine remains to be challenging. Here, a facile strategy is reported to prepare polydopamine (PDA)‐coated molybdenum disulfide (MoS_2_) nanoflakes as a nanocarrier to load dual drug cisplatin (Pt) and 1‐methyl‐tryptophan (1‐MT) for precision tumor theranostics. Preformed MoS_2_ nanoflakes are coated with PDA, modified with methoxy‐polyethylene glycol (PEG)‐amine, and loaded with 1‐MT and Pt. The formed functional 1‐MT‐Pt‐PPDA@MoS_2_ (the second P stands for PEG) complexes exhibit good colloidal stability and photothermal conversion efficiency (47.9%), dual pH‐, and photothermal‐sensitive drug release profile, and multimodal thermal, computed tomography and photoacoustic imaging capability. Due to the respective components of Pt, MoS_2_, and 1‐MT that can block the immune checkpoint associated to tumoral indoleamine 2,3‐dioxygenase‐induced tryptophan metabolism, tri‐mode chemo‐photothermo‐immunotherapy of tumors can be realized. In particular, under the near infrared laser irradiation, fast release of both drugs can be facilitated to achieve cooperative tumor therapy effect, and the combined immunogenic cell death induced by the dual‐mode chemo‐photothermo treatment and the 1‐MT‐induced immune checkpoint blockade can boost enhanced antitumor immune response to generate significant cytotoxic T cells for tumor killing. The developed 1‐MT‐Pt‐PPDA@MoS_2_ complexes may be used as an intelligent nanoplatform for cooperative precision imaging‐guided combinational tumor therapy.

## Graphical abstract

A multifunctional polydopamine‐coated molybdenum disulfide nanoplatform is developed to integrate a chemotherapy drug cisplatin and an immune checkpoint blocker 1‐methyl‐tryptophan (1‐MT). The designed functional complexes exhibit pH‐ and photothermal‐sensitive drug release profile and achieve cooperative multimode imaging‐guided tri‐mode chemo‐photothermo‐immunotherapy of tumors, where the immunotherapy is boosted by immune cell death/dendritic cell maturation‐induced cytotoxic T cells and the 1‐MT‐disrupted tryptophan metabolism.

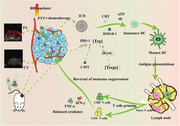

## 1 Introduction

At present, tumor resistance to multidrugs and tumor recurrence are major causes of patient death.^[^ ^1^ ^]^ Conventional treatments such as surgery, radiotherapy, and chemotherapy have their inherent limitations. In general, surgery cannot completely remove the tumor lesion boundary, and chemotherapy and radiotherapy are prone to cause great damages to surrounding normal tissues/organs and bring systematic toxic side effects. Developing a novel treatment strategy based on the emerging nanotechnology has been prevailing in the nanomedicine community for cancer therapy.^[^ ^2^ ^]^ In particular, nanoplatforms are able to integrate various imaging and therapeutic components for precision imaging‐guided therapy of different tumor types.^[^ ^3^ ^]^ In addition, nanoplatforms can be designed to have tumor microenvironment (such as acidic pH, glutathione, enzyme, or reactive oxygen species (ROS))‐responsive drug release profile to maximize the tumor therapy effect.^[^ ^4^ ^]^ Furthermore, various inorganic or organic–inorganic hybrid platforms with photothermal, catalytic, or ultrasound responsiveness have been developed to achieve photothermal therapy (PTT),^[^ ^5^ ^]^ chemodynamic therapy,^[^ ^6^ ^]^ or sonodynamic therapy.^[^ ^7^ ^]^ Importantly, by combination of an immunotherapeutic component, nanoplatforms can be designed to boost tumor immunotherapy.^[^ ^8^ ^]^

Among different therapeutic modes, immunotherapy is a new type of cancer treatment which includes the use of cytokines,^[^ ^9^ ^]^ development of cancer vaccines,^[^ ^10^, ^11^ ^]^ blockade of immune checkpoint,^[^ ^12^ ^]^ and T cell‐based adoptive immunotherapy.^[^ ^13^ ^]^ In particular, immune checkpoint blockade (ICB) therapy has received a great deal of attention for its long‐lasting immune responses. For instance, 1‐methyl‐tryptophan (1‐MT), an inhibitor of indoleamine 2,3‐dioxygenase (IDO), has been used to block the IDO‐catalyzed tryptophan (Trp) metabolism, thus enhancing the antitumor and antiviral immune responses of T cells.^[^ ^14^ ^]^ In this domain, Chen and co‐workers constructed injectable ROS‐responsive gels integrated with dual immune checkpoint blockers (1‐MT and programmed cell death‐1 antibody) for enhanced immunotherapy.^[^ ^15^ ^]^ However, immunotherapy alone is only effective to a small portion of cancer patients and may result in severe autoimmune‐associated side effects due to its broad specificity and dynamic nature of immune system. This has motivated the exploration of combined therapeutic strategy through the versatile nanotechnology.

PTT has been paid great attention in recent years for its noninvasive characteristics.^[^ ^16^ ^]^ In general, PTT based on the single use of photothermal agents is quite limited due to the low treatment efficiency and side effects in avoiding malignant tumor metastasis and recurrence.^[^ ^17^ ^]^ For effective cancer therapy, PTT has been combined with chemotherapy by integrating photothermal agents, such as gold nanostars,^[^ ^18^ ^]^ molybdenum disulfide nanoflakes (MoS_2_),^[^ ^19^ ^]^ or conducting polymers^[^ ^20^ ^]^ with chemotherapeutic drugs.^[^ ^21^ ^]^ For instance, in a recent study, Zhang and co‐workers developed polydopamine (PDA)‐coated gold nanorods that can be loaded with cisplatin (Pt) through covalent coordination for effective combination of PTT and chemotherapy.^[^ ^22^ ^]^ Nam and co‐workers demonstrate that PTT combined with chemotherapy can trigger potent antitumor immunity by polydopamine‐coated spiky gold nanoparticles (NPs) loaded with doxorubicin.^[^ ^23^ ^]^ The combination of PTT and chemotherapy can greatly improve the therapeutic effect of tumors at low doses to reduce the damage to normal tissues. Nonetheless, recent studies have revealed that the photothermo‐chemotherapy combination cannot effectively prevent the recurrence of malignant tumors due to the immune escape in the tumor microenvironment.^[^ ^24^ ^]^ Therefore, the combination of immunotherapy with chemotherapy and PTT may be an effective alternative to achieve a thorough treatment of tumors, which has not been systematically investigated.^[^ ^25^ ^]^

For cooperative tumor immunotherapy, it is vital to take the advantage of immunogenic cell death (ICD), which is beneficial to promote the maturation of dendritic cells (DCs) and the recognition and delivery of tumor antigens.^[^ ^26^ ^]^ It is known that PTT and/or chemotherapy can kill tumor cells effectively to generate the ICD.^[^ ^27^ ^]^ Meanwhile, immune checkpoint inhibitors can effectively relieve the immune suppression of the tumor microenvironment, contributing to the production of antitumor cytotoxic T cells. Therefore, it is essential to integrate external photothermo‐chemotherapy and the ICD or immune checkpoint inhibitors for combined therapy to significantly improve the tumor therapy outcome. For instance, Chen and co‐workers achieved the combined treatment of photothermo‐immunotherapy by constructing PDA‐coated Al_2_O_3_ NPs loaded with CpG.^[^ ^28^ ^]^ In a recent work, Dai and co‐workers prepared bioactive polyphenol nanoparticles (NPs) coordinated with Fe(II) ions for combined photodynamic therapy and chemotherapy, which can be further integrated with ICD and programmed cell death‐ligand 1 antibody to exert additional immunotherapy effect and immune responses to effectively inhibit tumor growth and metastasis.^[^ ^29^ ^]^ Tao and co‐workers developed a multifunctional platform using a self‐assembly strategy to incorporate materials with specific functions of chemotherapeutics, hyperthermia, and immunotherapy, which can collectively contribute to the effective cancer treatment.^[^ ^30^ ^]^ However, these nanomedicine platforms lack imaging capabilities for real time monitoring of the tumor treatment.

**Figure 1:**
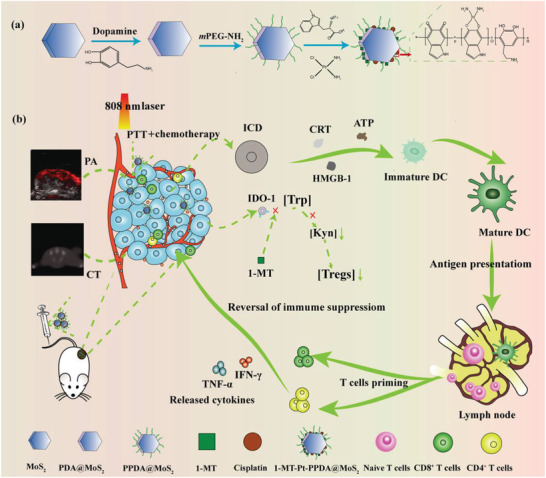
Schematic illustration of a) the synthesis of 1‐MT‐Pt‐PPDA@MoS_2_ for b) combined therapy of tumors.

To meet the challenges required in nanomedicine for multimode imaging‐guided combination of immunotherapy and other therapy modes, here we report the development of functional MoS_2_ nanoflakes as a new platform. In our work, MoS_2_ nanoflakes synthesized via a hydrothermal method were coated with PDA through polymerization under an aerobic alkaline condition, and loaded with 1‐MT and Pt through *π*–*π* interaction and covalently coordination, respectively (**Figure** **1**a). Considering the fact that certain chemotherapeutics would lead to IDO1 upregulation, it is rational to suppress IDO1 activity in combination with immunotherapy (e.g., 1‐MT) to achieve synergetic antitumor efficacy.^[^ ^31^ ^]^ The unique design systematically considers not only the cooperative multimode imaging through the elements of MoS_2_ nanoflakes and PDA for thermal, computed tomography (CT) and photoacoustic (PA) imaging, but also the multimode PTT/chemotherapy/immunotherapy through the MoS_2_ and the loaded Pt and 1‐MT, an IDO‐1 inhibitor. Strikingly, the design is expected to use IDO inhibitor to overcome the immune escape induced by chemotherapy and to reduce the side effects of chemotherapy through the thermo‐responsive release of Pt. We systematically investigated the structure, composition, morphology, stability, and photothermal conversion property of the hybrid NPs (1‐MT‐Pt‐PPDA@MoS_2_, the second P stands for polyethylene glycol, PEG), and explored their cooperative imaging and combined photothermo‐chemo‐immunotherapy of mouse melanoma cells (B16 cells) in vitro and the xenografted tumor model in vivo (Figure 1b). As far as we know, this is the very first case related to the development of functional MoS_2_ nanoflakes as a platform for combination of photothermo‐chemotherapy with the ICD/ICB‐based immunotherapy under multimode imaging guidance.

## 2 Results and Discussion

### 2.1 Synthesis and Characterization of 1‐MT‐Pt‐PPDA@MoS_2_ Complexes

In our work, we chose to synthesize MoS_2_ nanoflakes because they are biocompatible and display a high photothermal conversion efficiency for photothermal/PA imaging and PTT applications. MoS_2_ nanoflakes were synthesized via a known hydrothermal method reported in the literature.^[^ ^32^ ^]^ The formed MoS_2_ nanoflakes show an obvious near infrared (NIR) absorption feature (Figure S1, Supporting Information). After surface PDA coating, the NIR absorbance feature of the PDA@MoS_2_ complexes is enhanced due to the NIR absorption of PDA (Figure S1, Supporting Information), suggesting the successful surface modification of PDA. The grafting efficiency of PDA onto the MoS_2_ nanoflakes was calculated to be 13.0% based on the quantitative inductively coupled plasma‐optical emission spectroscopy (ICP‐OES) assay.

**Figure 2:**
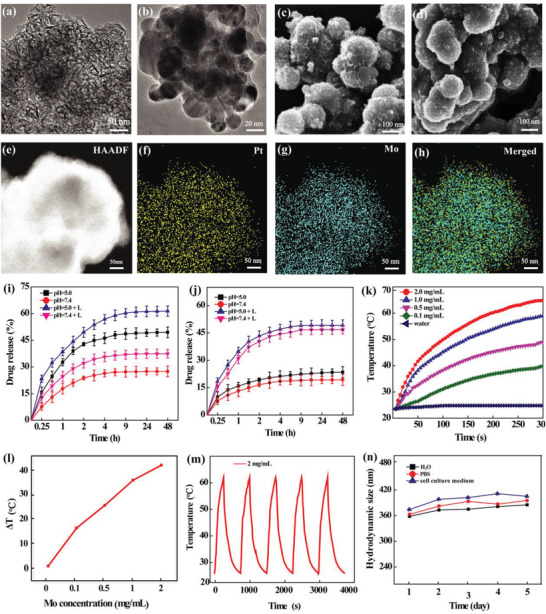
TEM images of a) MoS_2_ and b) PDA@MoS_2_ particles. SEM images of c) MoS_2_ and d) PDA@MoS_2_ particles. e) A high‐angle annular dark field (HAADF) TEM image of 1‐MT‐Pt‐PPDA@MoS_2_ and element mapping of Pt f), Mo g), and merged Pt and Mo h) for the 1‐MT‐Pt‐PPDA@MoS_2_ complexes. Cumulative release profile of i) Pt and j) 1‐MT from the 1‐MT‐Pt‐PPDA@MoS_2_ complexes at pH 5.0 and 7.4 with or without an 808 nm laser irradiation (1 W cm^−2^, 5 min) for each time point (*n* = 3). k) Temperature elevation plot of pure water and the aqueous suspension of 1‐MT‐Pt‐PPDA@MoS_2_ complexes at different Mo concentrations (0.1, 0.5, 1.0, and 2.0 mg mL^−1^, respectively) under an 808 nm laser irradiation (1 W cm^−2^, 5 min). l) Temperature change (Δ*T*) of the aqueous suspension of the 1‐MT‐Pt‐PPDA@MoS_2_ complexes under laser irradiation for 5 min as a function of Mo concentration. m) Temperature plot of the aqueous suspension of 1‐MT‐Pt‐PPDA@MoS_2_ complexes ([Mo] = 2 mg mL^−1^) during five cycles of laser on‐off (laser on: 300 s; laser off: cool to room temperature). n) Hydrodynamic size of the 1‐MT‐Pt‐PPDA@MoS_2_ complexes dispersed in water, PBS and cell culture medium as a function of storage time period (*n* = 3).

The morphology and size of MoS_2_ nanoflakes before and after PDA coating were observed by transmission electron microscopy (TEM, **Figure** **2**a,b) and scanning electron microscopy (SEM, Figure 2c,d). Obviously, the sharp flake morphology of MoS_2_ changes to be smooth to some extent after the surface modification of PDA. The size of the pristine MoS_2_ nanoflakes was measured to be about 25.0 nm from TEM images (Figure 2a) since the transparent TEM images allow one to differentiate the single particle boundary structure, whereas it is difficult to calculate the size of them through SEM imaging due to the surface sputter coating of the SEM sample and the aggregation of the flakes after the drying process (Figure 2c). After PDA coating, the aggregated flakes were closely combined to form tightly packed particles with an average diameter of about 150 nm, which can also be observed through SEM imaging (Figure 2d). Furthermore, energy dispersive spectroscopy (EDS) analysis confirmed the composition of Mo and S elements in the PDA@MoS_2_ hybrids (Figure S2, Supporting Information).

The hydrodynamic size and surface potential of MoS_2_ and PDA@MoS_2_ were also measured (Table S1, Supporting Information). The hydrodynamic size of MoS_2_ nanoflakes before and after PDA modification is 623.6 ± 2.5 and 471.0 ± 4.5 nm, respectively. This suggests that the PDA modification helps to decrease the aggregation tendency of MoS_2_ nanoflakes. Likewise, the negatively charged MoS_2_ nanoflakes (−40.3 ± 2.3 mV) increases to −32.5 ± 3.4 mV after PDA coating. Further Fourier transform infrared (FTIR) spectral analysis also confirmed the PDA coating of MoS_2_ nanoflakes (Figure S3, Supporting Information). The strong band at 1615 cm^−1^ is due to the structure of PDA benzene ring. In addition, the peaks at 1083 and 872 cm^−1^ are mainly attributed to the MoS_2_ component, in agreement with the literature_._^[^ ^32^ ^]^

In order to improve the biocompatibility and in vivo circulation behavior of PDA@MoS_2_, the hybrids were modified with methoxy‐PEG‐amine (*m*PEG‐NH_2_) through a Michael addition reaction between the amine group of *m*PEG‐NH_2_ and the PDA double bond under an alkaline condition (pH = 8.5). After PEGylation, the hydrodynamic size of the hybrids decreases to 268.9 ± 3.7 nm, while their surface potential increases to 5.53 ± 1.4 mV (Table S1, Supporting Information), implying that PEGylation enables enhanced stabilization of the hybrids by further decreasing their aggregation intendency. The PEGylation modification was also confirmed by FTIR spectra (Figure S3, Supporting Information), where the peaks at 1150–1060 and 1300–1260 cm^−1^ can be assigned to the ether bond and C─N bond, respectively, illustrating the successful *m*PEG conjugation.

Next, we loaded both 1‐MT and Pt onto the PEGylated PDA@MoS_2_ (PPDA@MoS_2_) through *π*–*π* stacking and covalent coordination bonding with PDA functional groups, respectively. 1‐MT is an indoleamine 2,3‐dioxygenase (IDO) inhibitor with an ability to prevent the conversion of Trp to kynurenine (Kyn), thus realizing disrupted Trp metabolism to suppress tumor regulatory T cells (Tregs) development.^[^ ^33^ ^]^ Pt is known as a chemotherapy drug that can kill tumor cells by inhibiting DNA replication and transcription in cancer cells. UV–vis spectrometry and ICP‐OES analysis reveal that the drug loading content (DL) (Table S2, Supporting Information) of Pt and 1‐MT are 32.1% and 55.8%, respectively, while the entrapment efficiency (EE) of them are 9.3% and 32.5%, respectively. This indicates the success of the dual drug loading. The existence of Pt along with the Mo and S elements within the PPDA@MoS_2_ was also confirmed by EDS element mapping (Figure 2f–h), where all three elements display the same distribution, indicating the uniform Pt loading for the finally formed 1‐MT‐Pt‐PPDA@MoS_2_ complexes. TEM imaging further shows that the morphology of the nanoflakes has not changed appreciably after drug loading (Figure S4, Supporting Information).

We next investigated the release kinetics of Pt and 1‐MT from the 1‐MT‐Pt‐PPDA@MoS_2_ complexes. Under an acidic condition (pH = 5.0), 49.36 ± 0.6% of the loaded Pt can be released from the 1‐MT‐Pt‐PPDA@MoS_2_ complexes at 48 h (Figure 2i), while only 28.56 ± 0.8% of the Pt is released at pH 7.4 at the same time point. This might be due to the protonation of both PDA catechol hydroxyl group and the Pt to form [Pt(H_2_O)_2_(NH_2_)_2_]^2+^, thus leading to the fast release of Pt, in agreement with the literature.^[^ ^22^ ^]^ Meanwhile, the release percentage of 1‐MT is 19.26 ± 0.7% at pH 7.4 at 48 h, and slightly increases to 23.46 ± 0.8% at pH 5.0 at the same time point due to its increased water solubility (Figure 2j). After that, we checked the impact of NIR laser irradiation‐induced photothermal heating on the release of both Pt and 1‐MT. Clearly, compared with the drug release without laser irradiation, the release rate of both drugs is significantly enhanced after laser irradiation. In particular, under laser irradiation, the release of 1‐MT can reach 49.22% and 46.81% under pH 5.0 and pH 7.4, respectively, at 48 h, while there are 61.38% and 37.41% of Pt released under the respective conditions at the same time point. These results suggest that the release of both drugs is pH‐dependent and can be further triggered by NIR laser irradiation. Taken into account the acidity of the tumor microenvironment, the fast drug release triggered by NIR laser irradiation should be beneficial for further enhanced tumor therapy.

The photothermal conversion performance of the 1‐MT‐Pt‐PPDA@MoS_2_ complexes was then evaluated. As expected, the temperature of the complex suspension increases with the increase of Mo concentration under an 808 nm laser irradiation (Figure 2k). By plotting the temperature change (Δ*T*) of the 1‐MT‐Pt‐PPDA@MoS_2_ complexes in aqueous solution as a function of Mo concentration (Figure 2l), we show that the Δ*T* at an Mo concentration of 0, 0.1, 0.5, 1, and 2 mg mL^−1^ is 1.5, 16.5, 25.5, 35.4, and 41.3 °C, respectively. To further demonstrate the photothermal stability of the 1‐MT‐Pt‐PPDA@MoS_2_ complexes, five cycles of NIR laser irradiation (laser on, 1 W cm^−2^, 5 min) and cooling down (laser off) of the particle suspension were recorded continuously at an Mo concentration of 2 mg mL^−1^ (Figure 2m). Apparently, there is no obvious change during the laser on‐off process, demonstrating their favorable photothermal stability and promising potential as a PTT agent. The photothermal conversion efficiency (*η*) of the 1‐MT‐Pt‐PPDA@MoS_2_ complexes and the drug‐free PPDA@MoS_2_ ([Mo] = 2 mg mL^−1^) was measured to be 47.9% and 48.5%, respectively (Figure S5, Supporting Information), suggesting that the drug loading does not appreciably alter their photothermal conversion efficiency. The colloidal stability of the 1‐MT‐Pt‐PPDA@MoS_2_ complexes dispersed in different aqueous media (H_2_O, PBS, or RPMI 1640 medium with 10% fetal bovine serum) was investigated through measuring their hydrodynamic size for 5 days. Obviously, the hydrodynamic size of the particles does not show any significant changes within a given time period (Figure 2n), thus validating their good colloidal stability.

### 2.2 Cellular Uptake and Cytotoxicity Assays

To exert the therapeutic functionality, it is essential for the 1‐MT‐Pt‐PPDA@MoS_2_ complexes to be taken up by cancer cells. We checked the cellular uptake of the 1‐MT‐Pt‐PPDA@MoS_2_ complexes by measuring the concentration of Mo and Pt with the B16 cells after the cells were treated with the complexes at different Mo concentrations for 6 h. ICP‐OES data reveal that the cellular uptake of both Mo and Pt is concentration‐dependent, and the uptake amount of Mo/Pt can reach 34.23 and 16.35 pg per cell, respectively, at the maximum Mo concentration (50 µg mL^−1^) tested (Figure S6, Supporting Information).

**Figure 3:**
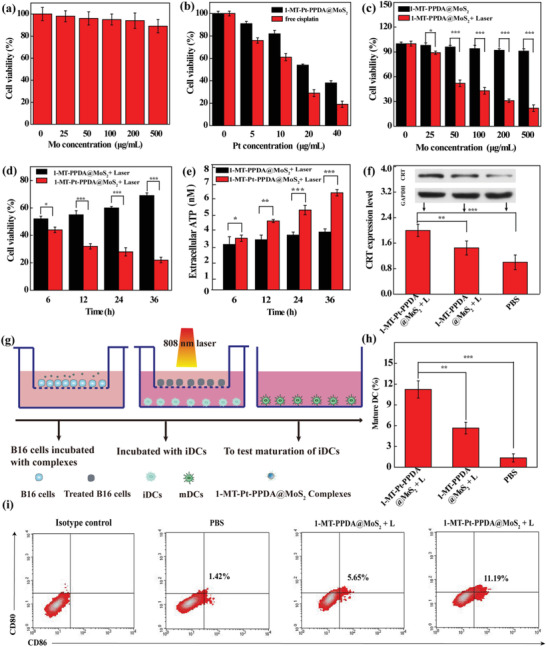
a) Viability of B16 cells treated with drug‐free PPDA@MoS_2_ for 24 h. b) Viability of B16 cells treated with free Pt or 1‐MT‐Pt‐PPDA@MoS_2_ complexes at various Pt concentrations for 24 h. c) Viability of B16 cells treated with the 1‐MT‐PPDA@MoS_2_ complexes at different concentrations for 24 h with or without laser irradiation (1 W cm^−2^, 5 min) treatment. d) Viability of B16 cells at different incubation durations after laser irradiation. e) Extracellular secretion of ATP from B16 cells at different incubation durations after laser irradiation. f) Western blot analysis of CRT expression on B16 cells after laser irradiation (1 W cm^−2^, 5 min) and further incubation for 36 h. g) Schematic illustration of the coculture system of B16 cells and iDCs. h) Quantification and i) flow cytometric analysis of DC maturation after 24 h coculture with ICD cancer cells generated through different treatments. In a–f,h), *n* = 3 for each sample, and *, **, and *** represent *p* < 0.05, *p* < 0.01, and *p* < 0.001, respectively.

Then, we first checked the cytotoxicity of drug‐free PPDA@MoS_2_ by Cell Counting Kit‐8 (CCK‐8) assay of the B16 cell viability. It can be seen that after 24 h incubation, the cytotoxicity of PPDA@MoS_2_ is extremely low, and the cell death is marginal even after the cells were incubated with the PPDA@MoS_2_ at an Mo concentration of 500 µg mL^−1^ (**Figure** **3**a). This means that the drug‐free PPDA@MoS_2_ particles possess good cytocompatibility. Next, we tested the chemotherapeutic effect of the dual drug‐loaded 1‐MT‐Pt‐PPDA@MoS_2_ complexes. Free Pt with the same Pt concentrations was also tested for comparison. Due to the biocompatibility of 1‐MT,^[^ ^15^ ^]^ we did not test free 1‐MT. Obviously, the cell viability decreases with the increase of Pt concentration for both the complexes and free Pt, suggesting that the loaded Pt within the complexes has a chemotherapeutic effect on the cancer cells. The viability of B16 cells reaches 38 ± 1.3% at 24 h after the cells were treated with the complexes at a Pt concentration of 40 µg mL^−1^ (Figure 3b). The half‐maximum inhibitory concentrations (IC_50_s) of the 1‐MT‐Pt‐PPDA@MoS_2_ complexes and free Pt were calculated to be 22.13 and 12.35 µg mL^−1^, respectively. The higher IC_50_ of the complexes than that of free Pt should be due to the fact that thePt has to be released from the complexes to exert its therapeutic functionality, and the concentration of Pt for both materials at the same time point should be different.

To study the in vitro photothermal treatment effect of the complexes, B16 cells were incubated with the Pt‐free 1‐MT‐PPDA@MoS_2_ complexes at different Mo concentrations for 24 h before and after laser irradiation. CCK‐8 assay reveals that in the absence of laser irradiation, the viability of B16 cells remains no significant change even at the highest Mo concentration tested (Figure 3c). In sharp contrast, their viability significantly decreases with the increase of Mo concentration under laser irradiation (*p* < 0.05 for the Mo concentration of 25 µg mL^−1^, and *p* < 0.001 for other concentrations above 25 µg mL^−1^). The cell viability retains 22% at the highest Mo concentration (500 µg mL^−1^) tested. To further confirm the PTT efficacy of the Pt‐free 1‐MT‐PPDA@MoS_2_ complexes, qualitative live‐dead staining of cancer cells with acetoxy methylester and propidium iodide was also performed after the cells were treated with the complexes at an Mo concentration of 50 µg mL^−1^ under laser irradiation (Figure S7, Supporting Information). It can be seen that under laser irradiation, cells treated with PBS are still viable and healthy, while cells treated with the complexes show obvious red fluorescent signals, indicating a significant portion of cell death. These results demonstrate that the Pt‐free 1‐MT‐PPDA@MoS_2_ complexes are able to exert apparent PTT effect of cancer cells due to the presence of the MoS_2_ nanoflakes with an NIR absorption feature.

In order to investigate the enhancing effect of the Pt‐containing 1‐MT‐Pt‐PPDA@MoS_2_ complexes through combined chemo‐photothermotherapy, B16 cells were pretreated with the 1‐MT‐Pt‐PPDA@MoS_2_ complexes for 24 h at an Mo concentration of 50 µg mL^−1^ and then the cells were laser irradiated and further incubated for different time periods before CCK‐8 assay (Figure 3d). Pt‐free 1‐MT‐PPDA@MoS_2_ complexes were also tested for comparison. Obviously, at 6 h post laser irradiation, the viabilities of cells treated with the 1‐MT‐Pt‐PPDA@MoS_2_ and 1‐MT‐PPDA@MoS_2_ complexes are 44.47 ± 0.62% and 52.42 ± 0.34%, respectively, suggesting that the cells are mostly inhibited through PTT at the early hours. With the extension of incubation time, cells treated with the 1‐MT‐PPDA@MoS_2_ complexes show gradually increased viability from 52.42% (6 h) to 69.72% (36 h). In contrast, the viability of B16 cells treated with the 1‐MT‐Pt‐PPDA@MoS_2_ + laser continuously decreases and reaches 22 ± 0.3% at 36 h. These results indicate that through combinational PTT and chemotherapy, the 1‐MT‐Pt‐PPDA@MoS_2_ complexes enable enhanced cancer cell inhibition.

To check the immune response of the combined PTT and chemotherapy, we examined the characteristic markers of ICD in B16 cells treated according to the above schedule, namely the release of adenosine triphosphate (ATP) from cells and expression of calreticulin (CRT) on the cell surface, which can promote DCs to enter the tumor area and strengthen their recognition and phagocytosis of antigen of apoptotic tumor cells. Compared with the single‐mode PTT though the 1‐MT‐PPDA@MoS_2_ + laser, cells treated with 1‐MT‐Pt‐PPDA@MoS_2_ + laser display significantly increased time‐dependent ATP release at the same time points (*p* < 0.05, Figure 3e). Similarly, the expression of CRT in the 1‐MT‐Pt‐PPDA@MoS_2_ + laser group is significantly higher than that of the 1‐MT‐PPDA@MoS_2_ + laser and PBS groups (*p* < 0.01, Figure 3f). This indicates that combined PTT and chemotherapy of cancer cells treated with the 1‐MT‐Pt‐PPDA@MoS_2_ + laser enables enhanced ICD of cancer cells than single‐mode PTT through the treatment with the 1‐MT‐PPDA@MoS_2_ + laser. Furthermore, immunofluorescent imaging of B16 cells after the same treatments was also performed to check the CRT expression on the surface of cells (Figure S8, Supporting Information). As can be seen, compared to the PBS control group, both the treatments of 1‐MT‐PPDA@MoS_2_ + laser and 1‐MT‐Pt‐PPDA@MoS_2_ + laser can induce a significant CRT expression on the cell surface, and the combination photothermo‐chemotherapy treatment (1‐MT‐Pt‐PPDA@MoS_2_ + laser) leads to a more significant expression of CRT than the single photothermal treatment (1‐MT‐PPDA@MoS_2_ + laser). Our data suggest that CRT of the cancer cells can transport from the endoplasmic reticulum to the surface of the cancer cells.

To examine if the ICD of cancer cells promotes the maturation of DCs, a specialized antigen‐presenting immune cells that can regulate T‐cell‐mediated immune responses to destroy the tumors, B16 cells treated with the 1‐MT‐Pt‐PPDA@MoS_2_ + laser were then incubated with immature DCs (iDCs) through a transwell system. The B16 cells in upper wells were treated with the 1‐MT‐Pt‐PPDA@MoS_2_ + laser and iDCs seeded in the bottom wells (receptor wells) were cultured together for 24 h (Figure 3g). Then, DCs were analyzed by flow cytometry to check the expression of costimulatory molecules of CD86 and CD80, typical markers of DC maturation (Figure 3h,i). Apparently, B16 cells treated with the 1‐MT‐Pt‐PPDA@MoS_2_ + laser display much more significant increase of the DC maturity than the single‐mode PTT and PBS groups (*p* < 0.01). This means that through combinational PTT and chemotherapy, the ICD of cancer cells enables the promotion of DC maturation, which is essential for subsequent T cell‐based tumor immunotherapy.

### 2.3 In Vitro and In Vivo CT, PA, and Thermal Imaging

The CT and PA imaging performances of the 1‐MT‐Pt‐PPDA@MoS_2_ complexes were first investigated through phantom studies (Figure S9, Supporting Information). As shown in Figure S9a (Supporting Information), the brightness of CT images and the corresponding HU value of the complexes increase with the Mo concentration. Similarly, the 1‐MT‐Pt‐PPDA@MoS_2_ complexes also display Mo concentration‐dependent PA imaging contrast enhancement due to the near‐infrared absorption characteristics of the MoS_2_ nanoflakes (Figure S9b, Supporting Information). Moreover, both the CT and PA values exhibit an excellent linear relationship as a function of Mo concentration. This implies that the developed complexes possess good CT/PA imaging properties.

**Figure 4:**
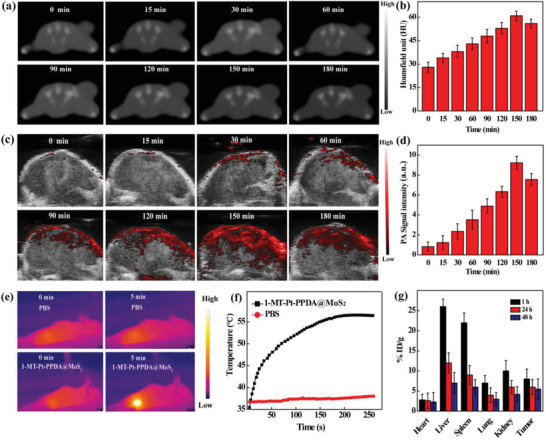
a) CT images and b) corresponding HU values of B16 tumors before and at different time points postinjection of the 1‐MT‐Pt‐PPDA@MoS_2_ complexes ([Mo] = 8 mg mL^−1^, in 0.1 mL PBS, *n* = 3 for each sample). c) PA images and d) corresponding PA values of tumors before and at different time points postinjection of the 1‐MT‐Pt‐PPDA@MoS_2_ complexes ([Mo] = 8 mg mL^−1^, in 0.1 mL PBS, *n* = 3 for each sample). e) Photothermal images and f) temperature change of tumors after intratumoral injection of the 1‐MT‐Pt‐PPDA@MoS_2_ complexes ([Mo] = 2 mg mL^−1^, in 0.1 mL PBS), followed by laser irradiation for 300 s. g) Time‐dependent Mo biodistribution in major organs of tumor‐bearing mice after intravenous injection of the 1‐MT‐Pt‐PPDA@MoS_2_ complexes ([Mo] = 8 mg mL^−1^, in 0.1 mL PBS, *n* = 3 for each sample). All doses used were just for each mouse.

Then, we explored the application of 1‐MT‐Pt‐PPDA@MoS_2_ complexes for multimodal CT/PA/thermal imaging of a subcutaneous B16 tumor model. Obviously, the CT value of tumor reaches the peak (61 HU) at 150 min postintravenous injection, which is about 2.2 times higher than that before injection (28 HU), and then starts to decrease due to the metabolization process (**Figure** **4**a,b). Similarly, the PA value of tumor reaches the peak (9.3) after intravenous injection of the 1‐MT‐Pt‐PPDA@MoS_2_ complexes. The coincidence of tumor CT/PA imaging data indicates the good in vivo stability of the complexes. Our data imply that the 1‐MT‐Pt‐PPDA@MoS_2_ complexes are able to be accumulated to the tumor site, likely attributed to the passive enhanced permeability and retention (EPR) effect.

In addition, due to the photothermal conversion effect, we tested the use of the 1‐MT‐Pt‐PPDA@MoS_2_ complexes for thermal imaging of tumors after intratumoral injection (Figure 4e). The temperature of the tumor region increases to 61 °C under an 808 nm laser irradiation for 300 s, while minimum temperature change is found in the tumor area treated with PBS under the same conditions (Figure 4f). Overall, the 1‐MT‐Pt‐PPDA@MoS_2_ complexes may act as a contrast agent for multimodal CT/PA/thermal imaging of tumors in vivo. Additionally, these three imaging modes are complementary: CT/PA imaging help to determine the optimal time point of the complexes accumulated to the tumor region for subsequent thermal imaging and PTT of tumors. In order to ensure the best therapeutic effect, we finally chose 2.5 h postinjection to perform the in vivo combination therapy of tumors.

To check the time‐dependent Mo distribution in tumor‐bearing mice, ICP‐OES analysis was performed by measuring the percentage of injected dose per gram of tissue (%ID/g, Figure 4g). At 1 h postinjection, a large quantity of Mo is taken up by the liver and spleen, followed by kidney and tumor. With the time postinjection, the uptake of Mo in all organs and tumors gradually decreases. This suggests that the complexes can be first cleared by reticuloendothelial system‐rich organs, accumulated to tumor site through EPR effect, and gradually metabolized and cleared out of body. Therefore, the developed 1‐MT‐Pt‐PPDA@MoS_2_ complexes possess a good biosafety profile.

### 2.4 Cooperative Thermo‐Chemo‐Immunotherapy of Tumors In Vivo

**Figure 5:**
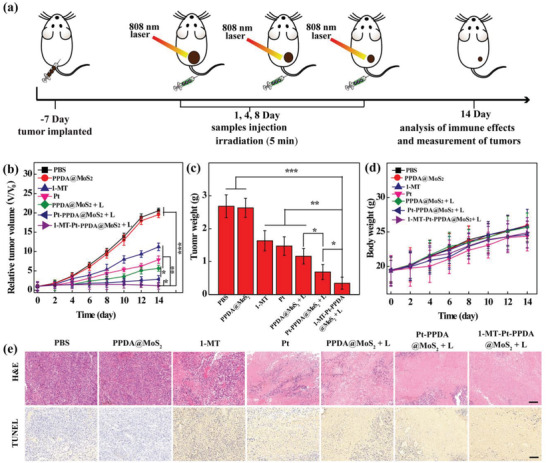
a) Schematic illustration and timeline of the combined therapy using the 1‐MT‐Pt‐PPDA@MoS_2_ complexes. b) Relative tumor volume as a function of time after different treatments for 14 days. c) Tumor weights dissected from different groups after the mice were treated for 14 days. d) Body weight changes of B16 tumor‐bearing mice as a function of time after different treatments. e) Hematoxylin & eosin (H&E) and terminal deoxynucleotidyl transferase‐mediated dUTP‐biotin nick end labeling (TUNEL)‐stained tumor slices from different groups after treatment for 14 days. Scale bar represents 100 µm for each panel. In b–d), *n* = 6 for each sample, and *, **, and *** represent *p* < 0.05, *p* < 0.01, and *p* < 0.001, respectively.

Next, we applied the 1‐MT‐Pt‐PPDA@MoS_2_ complexes for combinational chemotherapy, photothermal therapy, and immunotherapy of a subcutaneous tumor model. Shown in **Figure** **5**a is the tumor treatment schedule. Obviously, the tumors in the PBS and PPDA@MoS_2_ groups grow fast with the time postinjection (Figure 5b). In contrast, the treatment of free 1‐MT, free Pt, and drug‐free PPDA@MoS_2_ + laser shows moderate tumor growth inhibition effect (Figure 5b,c) in an order of PPDA@MoS_2_ + laser > Pt > 1‐MT, verifying the role played by single drugs or PTT. Strikingly, combined thermo‐chemotherapy (Pt‐PPDA@MoS_2_ + laser) and tri‐mode thermo‐chemo‐immunotherapy (1MT‐Pt‐PPDA@MoS_2_ + laser) enable much better tumor treatment efficiency than all the other groups (Figure 5b,c). Furthermore, the tri‐mode therapy displays a more significant tumor therapy effect than the dual‐mode therapy (*p* < 0.05). The enhanced tumor therapy effect could be due to the fact that the NIR laser irradiation can damage and even kill tumor cells, while the Pt drug can inhibit the proliferation and self‐repair of the damaged cancer cells. By the combination of an immune drug 1‐MT, the treatment of 1‐MT‐Pt‐PPDA@MoS_2_ + laser leads to complete ablation and eradication of tumors after 8 days of treatment, and the tumors do not recur during the experiment (Figure 5b,c). This might be due to the fact that the 1‐MT can effectively relieve the immune escape of the tumor microenvironment and take the advantages of the ICD of tumor cells induced by dual‐mode thermo‐chemotherapy to realize effective T cell‐based tumor immunotherapy. Additionally, the body weights of the treated mice in all groups exhibit a steady growth throughout the therapeutic process (Figure 5d), implying that there are negligible side effects exerted by all the different treatments.

H&E or TUNEL staining results show that the number of necrotic and apoptotic cells in the PBS and PDA@MoS_2_ groups is marginal, and the necrotic and apoptotic cell populations in the tumor slices follow the order of free 1‐MT < free Pt < PPDA@MoS_2_ + laser < Pt‐PPDA@MoS_2_ + laser < 1‐MT‐Pt‐PPDA@MoS_2_ + laser (Figure 5e). Especially, for the TUNEL staining, the cell apoptosis rate can be further quantified to prove the tumor inhibition efficacy (Figure S10, Supporting Information). Furthermore, H&E staining of major organs of tumor‐bearing mice was also performed (Figure S11, Supporting Information). There is no obvious damage and inflammation infiltration in the main organs, including the liver, lung, kidney, spleen, and heart, indicating the safety of all treatment types and the negligible side effects during the treatments. Compared to the results reported in the literature,^[^ ^22^ ^]^ our strategy concerns the use of multimode imaging guidance for precision treatment, and the treatment efficacy is better due to the combined mode of immunotherapy. Furthermore, the material preparation is simple, the cost is low, and the biological safety is high, which is more conducive for clinical translation.

### 2.5 In Vivo Immunotherapy Verification

**Figure 6:**
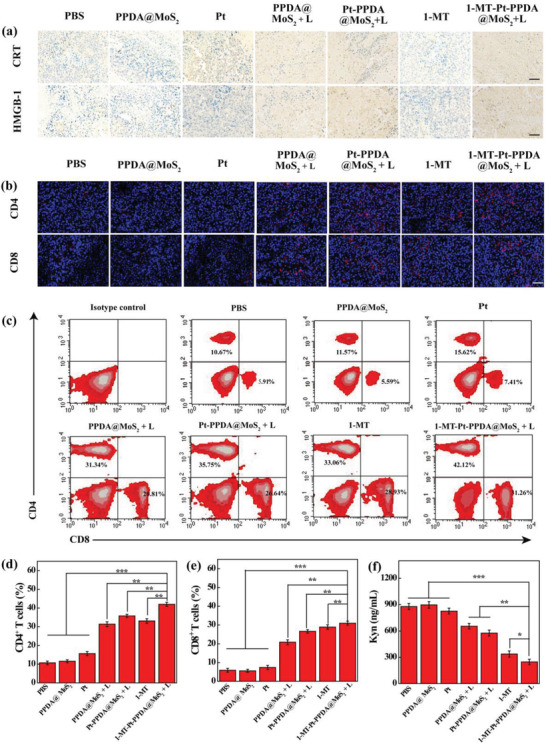
a) Immunohistochemical analysis of CRT and HMGB‐1 in tumor tissue. b) Representative immunofluorescence staining of CD4^+^ and CD8^+^ T cells in tumor tissue. Scale bar for each panel in a) and b) represents 100 µm . c–e) Flow cytometric examination of the intratumor infiltration of CD4^+^ and CD8^+^ T cells (CD4^+^ and CD8^+^ T cells were gated on CD3^+^ T cells). f) Kyn levels in the serum after various treatments. In d–f), *n* = 3 for each sample, and *, **, and *** represent *p* < 0.05, *p* < 0.01, and *p* < 0.001, respectively.

As mentioned above, the 1‐MT drug along with the ICD of tumor cells induced by thermo‐chemotherapy treatment may promote T cell‐based tumor immunotherapy. Here, we did immunohistochemical staining assay of the ICD marker expression in tumors after different treatments, including CRT and high mobility group protein 1 (HMGB‐1). When compared to PBS, PPDA@MoS_2_, and free 1‐MT groups, the treatments of 1‐MT‐Pt‐PPDA@MoS_2_ + laser and Pt‐PPDA@MoS_2_ + laser induce a significant CRT expression (**Figure** **6**a), followed by PPDA@MoS_2_ + laser and free Pt treatments. Similarly, consistent results can be seen for the expression of HMGB‐1 (Figure 6a), suggesting the enhanced tumor antigen presentation that could possibly promote DC maturation.

Next, in order to evaluate the possible ICD/DC maturation‐induced activation of T cells after the tri‐mode thermal‐chemo‐immunotherapy, the distribution of stimulated T cells in the spleens and tumors was examined through qualitative immunofluorescence staining of tumors (Figure 6b) and spleens (Figure S12, Supporting Information). For both tumor and spleen regions, the CD4^+^ and CD8^+^ T cells in the group of 1‐MT‐Pt‐PPDA@MoS_2_ + laser have the largest distribution among all groups, followed by the Pt‐PPDA@MoS_2_ + laser, 1‐MT, PPDA@MoS_2_ + laser, and free Pt groups. To quantify the phenotype of T cells in the tumor site by flow cytometry assay, we extracted the T cells and checked their purity through flow cytometric assay of cells bound with the fluorescein isothiocyanate‐labeled CD3 antibody (Figure S13, Supporting Information). The purity of T cells was determined to be 93.9%, which is essential for subsequent quantitative flow cytometry assay (Figure 6c–e). The populations of CD4^+^ and CD8^+^ T cells after different treatments of tumors follow the order of 1‐MT‐Pt‐PPDA@MoS_2_ + laser (42.12% and 31.26%) > Pt‐PPDA@MoS_2_ + laser (35.75% and 26.64%) ≈ free 1‐MT (33.06% and 28.93%) > PPDA@MoS_2_ + laser (31.34% and 20.81%) > free Pt (15.62% and 7.41%) > PPDA@MoS_2_ (11.57% and 5.59%) > PBS (10.67% and 5.91%). It should be noted that the relatively high populations of CD4^+^ T cells and CD8^+^ T cells in the group of free 1‐MT (33.06% and 28.93%) should be due to the inhibition of the Kyn, which was proven by detecting the content of Kyn in serum (Figure 6f). The populations of CD4^+^ T cells and CD8^+^ T cells in the tri‐mode therapy group of 1‐MT‐Pt‐PPDA@MoS_2_ + laser are 3.9 and 5.3 folds higher than those in the PBS group and obvious enhancement are also observed when compared to other groups (*p* < 0.01). This suggests that the cytotoxic T cells have effective tumor infiltration to exert their immunotherapy effect. These results are consistent with the above immunofluorescence staining of tumors (Figure 6b).

Tregs play an important role in the immune escape of tumor microenvironment. It is crucial to decrease the population of Tregs to realize effective tumor immunotherapy. We next tested the population of Tregs by immunofluorescence staining of Foxp3 protein, an important marker of Tregs in tumors (Figure S14, Supporting Information). The expression of Foxp3 in different tumor treatment groups is in an order of 1‐MT‐Pt‐PPDA@MoS_2_ + laser < free 1‐MT < Pt‐PPDA@MoS_2_ + laser < PPDA@MoS_2_ + laser < free Pt < PPDA@MoS_2_ < PBS. Clearly, due to the combination of the 1‐MT‐induced inhibition of Kyn and the ICD induced by thermo‐chemotherapy, significant inhibition of Tregs can be realized.

Moreover, to investigate the effective T cell‐mediated tumor immunotherapy, enzyme‐linked immunosorbent assay was carried out to determine the levels of tumor necrosis factor *α* (TNF‐*α*), interferon‐*γ* (IFN‐*γ*), and interleukin 6 (IL‐6) in the serum of mice after different treatments (Figure S15, Supporting Information). Apparently, the levels of TNF‐*α* (Figure S15a, Supporting Information), IFN‐*γ* (Figure S15b, Supporting Information), and IL‐6 (Figure S15c, Supporting Information) are in an order of 1‐MT‐Pt‐PPDA@MoS_2_+ laser > Pt‐PPDA@MoS_2_ + laser > free 1‐MT > PPDA@MoS_2_ + laser > free Pt > PPDA@MoS_2_ ≈ PBS. The highest expression of these cytokines after the treatment of 1‐MT‐Pt‐PPDA@MoS_2_+ laser further indicates the cooperative enhancement of tumor inhibition through tri‐mode therapy to lead to the best T cell‐mediated antitumor immune responses. It should be noted that compared with dual‐mode combination therapy that needs a relatively high dose of chemodrugs,^[^ ^34^ ^]^ tri‐modal chemo‐photothermo‐immunotherapy could achieve a desired therapeutic efficiency and synergy, and further lower the dosage of chemodrugs to reduce the side effects of chemotherapy. Furthermore, the photothermal agents (PDA and MoS_2_) caused PTT and ICD effects, the 1‐MT‐mediated immunotherapy could effectively suppress the high expression of IDO‐1 caused by chemotherapy, and the PTT‐ and chemotherapy‐induced ICD also facilitated immunotherapy.

## 3 Conclusions

In summary, we report the design of a dual drug‐loaded PDA‐coated MoS_2_ nanoplatform for cooperative multimodal imaging and combination therapy of tumors. We show that the hydrothermally synthesized MoS_2_ nanoflakes are able to be coated with PDA, PEGylated, and sequentially loaded with 1‐MT and Pt to create a stable functional nanomedicine platform, which displays good NIR absorption feature, X‐ray attenuation property, and pH‐dependent and NIR laser‐triggered release profile of both drugs. The developed 1‐MT‐Pt‐PPDA@MoS_2_ complexes are able to significantly inhibit the growth of cancer cells in vitro through the combined photothermo‐chemotherapy that can lead to the effective induction of ICD and DC maturation. These properties of the 1‐MT‐Pt‐PPDA@MoS_2_ complexes enabled multimode complementary CT/PA/thermo imaging of tumors. Furthermore, due to the cooperative ICD/DC maturation through photothermo‐chemotherapy and the 1‐MT‐resulted Kyn inhibition, active T cell‐mediated immunotherapy can be synchronized to realize complete tumor eradication. Overall, the prepared 1‐MT‐Pt‐PPDA@MoS_2_ complexes may represent one of the updated designs of nanomedicine platform for cooperative multimode imaging‐guided multimode therapy of different tumor types with an excellent treatment outcome.

## 4 Experimental Section

### Synthesis of PDA@MoS_2_ Complexes

MoS_2_ nanoflakes were synthesized according to the literature protocol.^[^ ^32^ ^]^ To coat PDA onto the surface of MoS_2_ nanoflakes, MoS_2_ nanoflakes (50 mg) were suspended in 50 mL of dopamine solution (1 mg mL^−1^, in water), which was adjusted to pH 8.5 using 10 × 10^−3^ m tris (hydroxymethyl) aminomethane buffer. The suspension was stirred for 24 h under an open air condition at room temperature, and then the complexes were collected by centrifugation (10 000 rpm, 15 min), and washed twice with water to create the PDA‐coated MoS_2_ complexes (for short, PDA@MoS_2_).

### Preparation of Multifunctional Dual Drug‐Loaded MoS_2_ Complexes

PDA@MoS_2_ hybrids were first modified with *m*PEG‐NH_2_ through a Michael addition reaction between the PDA surface double bonds and the PEG end amines under an alkaline condition (pH = 8.5). Typically, PDA@MoS_2_ particles (50 mg) were dispersed in 10 mL of aqueous *m*PEG‐NH_2_ solution (10 mg mL^−1^) under stirring. The solution was adjusted to pH 8.5 using 10 × 10^−3^ m tris (hydroxymethyl) aminomethane buffer. After incubation at 50 °C under stirring for 36 h, the complexes were retrieved by centrifugation (10 000 rpm, 10 min) and washed twice with water to get the product of PEGylated PDA@MoS_2_ (for short, PPDA@MoS_2_) particles.

For 1‐MT loading, a 1‐MT solution with a concentration of 0.25 mg mL^−1^ was prepared by dissolving it in water at a temperature of 50 °C. Then, the PPDA@MoS_2_ particles (50 mg) were suspended in 200 mL of the 1‐MT solution while stirring for 24 h at room temperature. Then, 50 mL of Pt solution (0.5 mg mL^−1^ in water) was added to the above suspension while stirring for additional 12 h at room temperature. Afterward, the dual drug‐loaded complexes (1‐MT‐Pt‐PPDA@MoS_2_) were collected by centrifugation (10 000 rpm, 10 min) and washed twice to achieve a final aqueous suspension that was stored at 4 °C before use.

### In Vitro Cell Culture Assays

B16 cells were regularly cultured and adopted for in vitro cellular uptake assay of 1‐MT‐Pt‐PPDA@MoS_2_ complexes, cytotoxicity assay of drug‐free PPDA@MoS_2_, evaluations of the chemotherapeutic effect of the 1‐MT‐Pt‐PPDA@MoS_2_ complexes, photothermal therapy effect of the Pt‐free 1‐MT‐PPDA@MoS_2_ complexes and combined chemo‐photothermotherapy effect of the dual drug‐loaded 1‐MT‐Pt‐PPDA@MoS_2_ complexes, the extracellular ATP level assay, and the CRT protein expression assay. DCs were regularly cultured and adopted for in vitro ICD‐induced immune activation assay.

### In Vivo Imaging and Tri‐Mode Chemo‐Photothermo‐Immunotherapy of Tumors

All animal experiments were carried out according to the protocols approved by the ethical committee of Donghua University for animal care and also in accordance with the policy of the National Ministry of Health. CT, PA, and thermal imaging of tumors, in vivo biodistribution of the complexes, in vivo antitumor therapeutic efficacy evaluation, histological examinations of tumors and major organs, immunohistochemistry staining of tumors and spleen, flow cytometric analysis of T cells extracted from the tumors, and quantification of cytokines in the serum were performed to examine the performances of tumor imaging, tumor tri‐mode chemo‐photothermo‐immunotherapy and immune responses after different treatments.

### Statistical Analysis

Data were presented as the means ± standard deviations (for all data, *n* ≥ 3). One‐way analysis of variance statistical method was adopted to analyze the experimental results using IBM SPSS Statistics 25 software. A *p* value of 0.05 was selected as the significance level, and the data were marked with (*) for *p* < 0.05, (**) for *p* < 0.01, and (***) for *p* < 0.001, respectively.

## Conflict of Interest

The authors declare no conflict of interest.

## Supporting information

Supporting Information

## Data Availability

Research data are not shared.
