# Supplementary material for: Intelligent Molybdenum Disulfide Complexes as a Platform for Cooperative Imaging‐Guided Tri‐Mode Chemo‐Photothermo‐Immunotherapy
Source: Adv Sci (Weinh). 2021 Jun 18;8(14):2100165. doi: 10.1002/advs.202100165 (PMC8292874; doi:10.1002/advs.202100165)
Supplement: Supplementary file 1 — Supporting Information [file ADVS-8-2100165-s001.pdf]

© 2021 Wiley-VCH GmbH

ADVANCED SCIENCE
Open Access

Supporting Information

for *Adv. Sci.,* DOI: 10.1002/advs.202100165

Intelligent Molybdenum Disulfide Complexes as a Platform for Cooperative Imaging-Guided Tri-mode Chemo-Photothermo-Immunotherapy

Wei Hu, Tingting Xiao, Du Li, Yu Fan, Lingxi Xing*, Xipeng Wang, Yulin Li*, Xiangyang Shi*, Mingwu Shen*

Supporting Information

Intelligent Molybdenum Disulfide Complexes as a Platform for Cooperative Imaging-Guided Tri-mode Chemo-Photothermo-Immunotherapy

Wei Hu, Tingting Xiao, Du Li, Yu Fan, Lingxi Xing, Xipeng Wang, Yulin Li*, Xiangyang Shi*, Mingwu Shen**

W. Hu, T. T. Xiao, D. Li, Y. Fan, Prof. X. Y. Shi, Prof. M. W. Shen
State Key Laboratory for Modification of Chemical Fibers and Polymer Materials, College of Chemistry, Chemical Engineering and Biotechnology, Donghua University, Shanghai 201620, People's Republic of China
E-mail: xshi@dhu.edu.cn (X. Y. Shi) and mwshen@dhu.edu.cn (M. W. Shen)

Dr. L. X. Xing, Prof. Xipeng Wang
Department of Gynecology and Obstetrics, XinHua Hospital Affiliated to Shanghai Jiao Tong University School of Medicine, Shanghai 200092, People' Republic of China
E-mail: Xing_Lingxi@163.com

Prof. Yulin Li
The Key Laboratory for Ultrafine Materials of Ministry of Education, State Key Laboratory of Bioreactor Engineering, Engineering Research Center for Biomedical Materials of Ministry of Education, School of Materials Science and Engineering, East China University of Science and Technology, Shanghai 200237, People' Republic of China
E-mail: yulinli@ecust.edu.cn

Prof. X. Y. Shi
CQM-Centro de Quimica da Madeira, Universidade da Madeira, 9020-105 Funchal, Portugal

Experimental Section

Materials: Ammonium tetrathiomolybdate ($(NH_4)_2MoS_4$), hydrazinium hydroxide ($(N_2H_4)\cdot H_2O$, 98%), 1-methyl-tryptophan (1-MT) and cisplatin were purchased from J&K China Chemical, Ltd. (Beijing, China). Methoxy-polyethylene glycol-amine (*m*PEG-NH_2, Mw = 2000) was from ToYongBio Tech., Inc. (Shanghai, China). Dopamine hydrochloride (DA·HCl) was from TCI Development Co., Ltd. (Shanghai, China). Calcein Acetoxy Methylester (AM) and Propidium Iodide (PI) were from KeyGEN BioTECH Co., Ltd. (Nanjing, China). All chemicals were of reagent grade and used as received. B16 cells (a murine melanoma cell line) were from Institute of Biochemistry and Cell Biology, the Chinese Academy of Sciences (Shanghai, China). Dendritic cells (DCs) were from Shanghai Cancer Center, Fudan University. Cell Counting Kit-8 (CCK-8), 4',6-diamidino-2-phenylindole dihydrochloride (DAPI) and ATP assay kit were acquired from Beyotime Institute of Biotechnology (Shanghai, China). RPMI 1640 medium, penicillin, streptomycin, and fetal bovine serum (FBS) were from Gibco (Carlsbad, CA). Tumor Infiltrating Lymphocyte Cell Separation Medium Kit, Mice was from Beijing Solarbio Science & Technology Co., Ltd. (Beijing, China). Anti-calreticulin (CRT) rabbit polyclonal antibody (pAb), anti-GAPDH rabbit pAb, anti-high mobility group protein 1 (HMGB-1) rabbit pAb, anti-CD4 rabbit pAb and anti-CD8 alpha rabbit pAb were from Servicebio (Wuhan, China). Anti-CD4-PE (phycoerythrin), anti-CD8-FITC (fluorescein isothiocyanate), anti-CD86 PE, anti-CD80 FITC, and IgG isotype control were from Thermo Fisher Scientific (Waltham, MA). Regenerated cellulose dialysis membrane with a molecular weight cut-off (MWCO) of 1000 was acquired from Fisher Scientific (Pittsburgh, PA). Water used in all experiments was purified using a PURIST UV Ultrapure Water System (RephiLe Bioscience, Ltd., Shanghai, China) with a resistivity higher than 18.2 MΩ·cm.

Synthesis of PDA@MoS_2 Complexes: MoS_2 nanoflakes were synthesized according to the literature protocol.[1] To coat polydopamine (PDA) onto the surface of MoS_2 nanoflakes, MoS_2 nanoflakes (50 mg) were suspended in 50 mL of dopamine solution (1 mg/mL, in water), which was adjusted to pH 8.5 using 10 mM tris (hydroxymethyl) aminomethane buffer. The suspension was

stirred for 24 h under an open air condition at room temperature, and then the complexes were collected by centrifugation (10000 rpm, 15 min), and washed twice with water to create the PDA-coated MoS_2 complexes (for short, PDA@MoS_2).

Preparation of Multifunctional Dual Drug-Loaded MoS_2 Complexes: PDA@MoS_2 hybrids were first modified with *m*PEG-NH_2 through a Michael addition reaction between the PDA surface double bonds and the PEG end amines under an alkaline condition (pH = 8.5). Typically, PDA@MoS_2 particles (50 mg) were dispersed in 10 mL of aqueous *m*PEG-NH_2 solution (10 mg/mL) under stirring. The solution was adjusted to pH 8.5 using 10 mM tris (hydroxymethyl) aminomethane buffer. After incubation at 50 ℃ under stirring for 36 h, the complexes were retrieved by centrifugation (10000 rpm, 10 min) and washed twice with water to get the product of PEGylated PDA@MoS_2 (for short, PPDA@MoS_2) particles.

For 1-MT loading, a 1-MT solution with a concentration of 0.25 mg/mL was prepared by dissolving it in water at a temperature of 50 ℃. Then, the PPDA@MoS_2 particles (50 mg) were suspended in 200 mL of the 1-MT solution while stirring for 24 h at room temperature. Then, 50 mL of cisplatin solution (0.5 mg/mL in water) was added to the above suspension while stirring for additional 12 h at room temperature. Afterwards, the dual drug-loaded complexes (1-MT-Pt-PPDA@MoS_2) were collected by centrifugation (10000 rpm, 10 min) and washed twice to achieve a final aqueous suspension that was stored at 4 ^{o}C before use. To quantify the loading of 1-MT and cisplatin, ninhydrin assay and inductively coupled plasma-optical emission spectroscopy (ICP-OES, Leeman Prodigy, Hudson, NH) were respectively employed to measure the merged supernatants after centrifugation and after additional two cycles of washing/centrifugation steps. The drug loading content (DL) and entrapment efficiency (EE) of 1-MT and cisplatin were calculated according to the following equations:

$$DL\ (\%) = M_t / M_0 \times 100\% \quad (1)$$

$$EE\ (\%) = M_t / M_L \times 100\% \quad (2)$$

where M_t, M_0, and M_L stand for the masses of encapsulated 1-MT or cisplatin, the initial 1-MT or cisplatin, and the drug loaded complexes, respectively.

Characterization Techniques: Zeta potential and hydrodynamic size were tested using a Malvern Zetasizer Nano ZS model ZEN3600 system (Worcestershire, UK) coupled with a standard 633-nm laser. Samples were dispersed in H_2O ([Mo] = 1.0 mg/mL) before measurements. UV-vis spectra were acquired using a Lambda 25 UV-vis spectrophotometer (Perkin Elmer, Waltham, MA) and samples were dispersed in H_2O ([Mo] = 0.1 mg/mL) before measurements. Fourier transform infrared (FTIR) spectra were recorded on a Nexus 670 spectrometer (Thermo Nicolet Corporation, Madison, WI). Samples were mixed with milled KBr crystals and pressed to form 13-mm diameter disks before measurements. Transmission electron microscopy (TEM) imaging was executed using a JEOL2010F analytical electron microscope (JEOL, Tokyo, Japan) at an operating voltage of 200 kV. Each sample dispersed in water ([Mo] = 1.0 mg/mL) was dropped onto a carbon-coated copper grid and air dried before measurements. An energy dispersive X-ray spectroscopy (EDS) detector coupled with the TEM instrument was used to analyze the elemental composition of samples. Scanning electron microscopy (SEM) imaging was executed using a S-4800 SEM (Hitachi, Ltd., Tokyo, Japan) at a voltage of 5 kV. Each sample was prepared by dropping an aqueous particle suspension ([Mo] = 1.0 mg/mL) onto an aluminum foil, air dried, and sputter coated with a gold film with a thickness of 10 nm before measurements. To study the colloidal stability of the 1-MT-Pt-PPDA@MoS_2, the particles were dispersed in H_2O, phosphate buffered saline (PBS) or cell culture medium (RPMI 1640 with 10% FBS) at an Mo concentration of 1.0 mg/mL (1 mL) to test their hydrodynamic size for five consecutive days.

Drug Release Kinetics: To study the release profile of the loaded 1-MT and cisplatin, the 1-MT-Pt-PPDA@MoS_2 complexes were suspended in 20 mL of phosphate buffer at different pHs (5.0 or 7.4). Typically, an aqueous solution of the 1-MT-Pt-PPDA@MoS_2 complexes (1 mg/mL, 2 mL) was placed in a dialysis bag (MWCO = 1000), and then exposed to 18 mL of PBS (pH 7.4) or phosphate buffer (pH 5.0). The entire system was kept in a constant temperature vibrator at 37 ℃ under gentle shaking. At different time intervals, 2 mL of solution was taken out from the outer phase and the volume of outer phase was maintained constant by adding 2 mL of the corresponding buffer solution. The amount of released 1-MT and cisplatin was measured *via* ninhydrin assay and

ICP-OES, respectively. To check if the NIR irradiation-induced photothermal heating could trigger the fast drug release from the complexes, the 1-MT-Pt-PPDA@MoS_2 complexes dispersed in pH 5.0 and pH 7.4 were irradiated by an 808-nm laser (Shanghai Xilong Optoelectronics Technology Co. Ltd., Shanghai, China, 1 W/cm^2, 5 min) at different time points (0.25, 0.5, 1, 1.5, 2, 3, 4, 6, 9, 12, 24, 36, and 48 h, respectively).

Photothermal Property of the 1-MT-Pt-PPDA@MoS_2 Complexes: The photothermal property of the dual drug-loaded complexes was assessed using an 808-nm laser. In brief, an aqueous suspension of the 1-MT-Pt-PPDA@MoS_2 complexes (0.2 mL) with different Mo concentrations (0.1, 0.5, 1.0, and 2.0 mg/mL, respectively) was put into a 0.5-mL Eppendorf tube (water was used as a negative control), followed by irradiation with an 808-nm laser at a power density of 1.0 W/cm^2 for 5 min. The temperature changes of different samples were recorded by an online DT-8891E thermocouple thermometer (Shenzhen Everbest Machinery Industry Co., Ltd., Shenzhen, China) every 5 s. Furthermore, the photothermal stability of the 1-MT-Pt-PPDA@MoS_2 complexes ([Mo] = 2.0 mg/mL) was tested by laser irradiation of the suspension with an 808-nm laser (1 W/cm^2 for 5 min) and cooling down to room temperature. The irradiation and cooling down processes were performed for 5 times.

Quantitative analysis of the photothermal conversion efficiency (η) of the 1-MT-Pt-PPDA@MoS_2 and PPDA@MoS_2 was carried out according to the literature.[1] In brief, the 1-MT-Pt-PPDA@MoS_2 or PPDA@MoS_2 dispersed in water (0.1 mL) with an Mo concentration of 2 mg/mL were placed in a 200-μL Eppendorf tube and irradiated by an 808-nm laser at 1.0 W/cm^2 for 5 min. The temperature of the aqueous solution of the 1-MT-Pt-PPDA@MoS_2 and PPDA@MoS_2 was monitored every 5 s using a thermocouple probe. The η was calculated by the following equation :

$$\eta = \frac{hS(T_{Max} - T_{Sur}) - Q_S}{I(1 - 10^{-A\lambda})} \tag{3}$$

where h is the heat transfer coefficient, S the surface area of the sample cuvette, T_{Max} the steady-state temperature, T_{Sur} the temperature of the surroundings, Q_S the heat associated with the

light absorbance of the solution, *I* the incident laser power, and A_λ the absorbance at a wavelength of 808 nm.

Computed Tomography (CT) and Photoacoustic (PA) Imaging Properties of the Complexes: To study the X-ray attenuation property, the 1-MT-Pt-PPDA@MoS_2 complexes with different Mo concentrations (1, 2, 3 and 4 mg/mL, respectively) were prepared in 0.5-mL Eppendorf tubes and the tubes were placed in a home-made scanning holder. Subsequently, CT scanning was performed using a dual-source SOMATOM Definition Flash CT system (Siemens, Erlangen, Germany) with 140 KV and a slice thickness of 0.6 mm. The evaluation of the X-ray attenuation intensity was carried out using a standard display program from the manufacturer. Contrast enhancement was determined in Hounsfield (HU) for each sample with different Mo concentrations.

The PA imaging property of the 1-MT-Pt-PPDA@MoS_2 complexes were checked by a Vevo LAZR system (Visual Sonics, Toronto, Ontario, Canada) with the following parameters: Frequency, 20 MHz; PA gain, 35 dB; and excitation wavelength, 808 nm. Before PA imaging, the 1-MT-Pt-PPDA@MoS_2 solution with different Mo concentrations (1, 2, 3 and 4 mg/mL, respectively) was injected into a thin tube embedded in the glue and then irradiated under an 808-nm laser. After that, the PA image was collected and the PA intensity of the sample at different Mo concentrations was quantified.

Cell Culture, Cellular Uptake and Cytotoxicity Assays: B16 cells were regularly cultured and passaged using RPMI 1640 medium supplemented with 10% FBS and 1% penicillin-streptomycin. The cells were cultured in a cell incubator at 37 ^{o}C and 5% CO_2.

To check the cellular uptake efficiency, B16 cells were seeded in a 12-well plate at a density of 1×10^5 cells per well in 1 mL of RPMI 1640 medium. After overnight incubation, the medium of each well was replaced with 1 mL of fresh medium containing different concentrations of 1-MT-Pt-PPDA@MoS_2 ([Mo] = 0, 2.5, 5, 10, 20 and 50 μg/mL, respectively) for 6 h. The cells were then rinsed three times with PBS and trypsinized. After counting the cell number in the cell suspensions, the cells were centrifuged to remove the supernatant, digested by *aqua regia* (1 mL),

and the solution was diluted to 5 mL. Then, ICP-OES was performed to determine the cellular Pt and Mo contents in the cell samples.

For cytotoxicity assay, we first evaluated the *in vitro* cytotoxicity of the drug-free complexes *via* a standard CCK-8 cell viability assay. B16 cells were seeded in a 96-well plate at a density of 1×10^4 cells per well in 0.1 mL medium and cultured overnight at 37 ^oC and 5% CO_2. Then, the cells in each well were treated with 0.1 mL medium containing PPDA@MoS_2 at different Mo concentrations ([Mo] = 0, 25, 50, 100, 200 and 500 μg/mL, respectively) for 24 h. Next, the cell viability was evaluated using the CCK-8 assay according to the manufacturer's instructions. After incubation, the culture medium was removed, and the cells were washed twice with PBS (pH = 7.4). Subsequently, 10 μL of CCK-8 assay reagent and 90 μL of fresh culture medium were mixed and added into each well and the cells were incubated for another 2 h under regular cell culture conditions. After that, the cells in each well were analyzed using a Thermo Scientific Multiskan MK3 enzyme-linked immunosorbent assay (ELISA) reader (Waltham, MA) at 450 nm.

Then, the complexes with drug payload (1-MT-Pt-PPDA@MoS_2) were tested to evaluate their *in vitro* chemotherapeutic effects. B16 cells were seeded in a 96-well plate at a density of 1×10^4 cells per well with 0.1 mL medium. After culturing overnight, the cells of each well were treated with 0.1 mL medium containing the 1-MT-Pt-PPDA@MoS_2 at different concentrations ([Pt] = 0, 5, 10, 20 and 40 μg/mL, respectively) for 24 h. Free cisplatin with the same concentrations was also tested for comparison. The cell viability was evaluated using CCK-8 assay according to the manufacturer's instructions.

To investigate the photothermal therapy (PTT) effect of the 1-MT-PPDA@MoS_2 complexes in the presence of NIR laser irradiation, CCK-8 cell viability and qualitative live-dead cell staining assays were performed. According to the above protocols used to check the cytotoxicity, B16 cells were treated with 1-MT-PPDA@MoS_2 at different concentrations ([Mo] = 0, 25, 50, 100, 200, and 500 μg/mL, respectively) for 24 h. After that, the cells in each well were washed twice with PBS (pH =7.4), incubated with 0.1 mL fresh culture medium, and laser irradiated with an 808-nm laser (1 W/cm^2) for 5 min. The cells without laser irradiation were used for comparison. The cells were then

incubated under 37 °C and 5% CO_2 for additional 3 h before CCK-8 assay according to the manufacturer's instruction. In parallel, the cells were treated similarly according to the above protocols, stained with calcein AM (2 μM) and PI (4 μM) indicative of the live (green) and dead (red) cells for 15 min at room temperature, and observed by an Axio Vert. A1 Carl Zeiss fluorescence microscopy (Jena, Germany).

***In Vitro* Combined Therapy and Extracellular ATP Level Determination:** To investigate the combined chemo-photothermal therapeutic effects of the drug-loaded 1-MT-PPDA@MoS_2 complexes *in vitro*, B16 cells were first seeded in 96-well plates and incubated with the 1-MT-Pt-PPDA@MoS_2 complexes ([Mo] = 50 μg/mL) for 24 h according to the above procedures. Cisplatin-free 1-MT-PPDA@MoS_2 complexes were used as control. After incubation, the cells were washed twice with PBS (pH =7.4), replenished with fresh culture medium, irradiated with an NIR laser (808-nm, 1 W/cm^2) for 5 min, and maintained at 37 °C and 5% CO_2 for different time periods (6, 12, 24 and 36 h, respectively). After that, the cell viabilities of different groups were evaluated through CCK-8 assay. Likewise, the cell culture medium was collected to measure the extracellular ATP level according to the literature protocols.[2] In brief, 0.1 mL of cell culture medium from each well was placed in each well of a 96-well black plate prefilled with an ATP detection working solution (0.1 mL) and gently vibrated. The luminescence (RLU) was recorded using a multifunctional microplate reader (BioTke Synergy 2, Winooski, VT).

Western Blot Assay *in Vitro*: To check the immunogenic cell death (ICD) of cancer cells after different treatments, B16 cells were seeded into 6-well plates at a density of 2×10^5 cells per well with 2 mL medium and cultured overnight at 37 °C and 5% CO_2. Then, the cells in each well were added with 2 mL medium containing 1-MT-PPDA@MoS_2 or 1-MT-Pt-PPDA@MoS_2 ([Mo] = 50 μg/mL). PBS was used as control. After incubation for 24 h, the cells were washed twice with PBS (pH =7.4), cultured with fresh culture medium, and treated with or without an NIR laser (808-nm, 1 W/cm^2) for 5 min. The cells were then cultivated for 36 h, trypsinated, collected in centrifuge tubes, and washed with PBS for three times. Then, the cells were centrifuged, added with 0.2 mL of lysis buffer containing phenylmethanesulfonyl fluoride, and incubated in an ice-bath for 30 min. After

that, the cell lysates were analyzed *via* Western blot assay to detect the CRT protein expression according to literature protocols.[3] GAPDH was used as a reference protein.

Immunofluorescent Staining of CRT *in Vitro*: To check the surface level of CRT on the tumor cells, B16 cells were seeded into confocal dishes at a density of 2 × 10^5 cells per dish with 1 mL medium and cultured overnight at 37 °C and 5% CO_2. Then, the cells in each dish were added with 1 mL medium containing 1-MT-PPDA@MoS_2 or 1-MT-Pt-PPDA@MoS_2 ([Mo] = 50 μg/mL, in 50 μL PBS). PBS was used as control. After incubation for 24 h, the cells were washed twice with PBS (pH =7.4), cultured with fresh medium, and treated with or without an NIR laser (808 nm, 1 W/cm^2) for 5 min. The cells were then cultivated for 24 h, washed with PBS for 2 times, and stained with anti-CRT primary antibody (0.1 mg/mL, in 2 μL PBS, and diluted with PBS to reach 1 mL) at 37 °C for 1.5 h. Then, the cells were incubated with Cy3-labeled secondary antibody. Finally, the cell nuclei were counter stained with DAPI, and the cells were observed using confocal laser scanning microscopy (Carl Zeiss LSM 700, Jena Germany).

***In Vitro* ICD-Induced Maturation of Dendritic Cells (DCs):** To investigate the *in vitro* ICD-induced immune activation of DCs, a 6-well transwell system with 0.4-μm polycarbonate porous membranes was used. Briefly, 1 × 10^5 B16 cells per well were seeded in the upper wells of the transwell system. After overnight culture to reach 65-75% confluence, B16 cells were incubated with 1-MT-Pt-PPDA@MoS_2 or 1-MT-PPDA@MoS_2 ([Mo] = 50 μg/mL) for 24 h. B16 cells treated with PBS were used as control. After incubation, the cells were washed twice with PBS (pH =7.4) and replenished with fresh culture medium. Afterward, the upper wells were merged with the lower wells for mixed culture of both B16 cells and DCs, which were seeded in the bottom wells at a density of 2 × 10^5 cells per well for 24 h. The upper wells were then treated with an NIR laser (808 nm, 1 W/cm^2) for 5 min. After incubation for 24 h, DCs were collected in 15-mL centrifuge tubes, and stained with IgG isotype control, or anti-CD86 PE/anti-CD80 FITC for 15 min. After centrifugation (1000 rpm, 5 min), DCs were resuspended in 0.2 mL of PBS and analyzed using a

FACS Calibur flow cytometer (Becton Dickinson, Franklin Lakes, NJ). Each sample were tested in triplicate.

***In Vivo* CT, PA and Thermal Imaging of Tumors:** All animal experiments were performed following the protocols approved by the ethical committee for animal care of Donghua University and also in accordance with the policy of the National Ministry of Health. To establish a xenografted tumor model, female 4 week-old C57BL/6 mice (Shanghai Slac Laboratory Animal Center, Shanghai, China) with an average weight of 20 g were subcutaneously injected with 1 × 10^6 B16 cells (in 0.1 mL of PBS, for each mouse) in the right back leg. When the tumor nodules reached a volume of about 100 mm^3, the mice were anesthetized by pentobarbital sodium (40 mg/kg for each mouse) *via* intraperitoneal injection before following experiments.

For CT imaging, 1-MT-Pt-PPDA@MoS_2 complexes dispersed in PBS ([Mo] = 8 mg/mL, 0.1 mL) were intravenously injected into each tumor-bearing mouse. CT images of the mice at different time points were collected by a dual-source SOMATOM Definition Flash CT system (Siemens, Erlangen, Germany) with the parameters similar to those mentioned above. The corresponding CT values were then quantified at 0, 15, 30, 60, 90, 120, 150, or 180 min post-injection.

For PA imaging, after intravenously injected with 1-MT-Pt-PPDA@MoS_2 ([Mo] =8 mg/mL, in 0.1 mL PBS for each mouse), the PA images and corresponding signal intensities of tumors were recorded at 0, 15, 30, 60, 90, 120, 150, or 180 min post-injection by the same Vevo LAZR system as described above.

In vivo thermal imaging of tumor-bearing mice was carried out by an infrared camera after intratumorally injected with 1-MT-Pt-PPDA@MoS_2 ([Mo] =2 mg/mL, in 0.1 mL PBS for each mouse). The mouse in the control group was injected with PBS (0.1 mL). After injection, the tumor tissue was exposed to an 808-nm laser (1 W/cm^2) for 5 min, and the thermal images and corresponding temperature values were captured.

***In Vivo* Biodistribution:** The 1-MT-Pt-PPDA@MoS_2 complexes ([Mo] = 8 mg/mL, in 0.1 mL of PBS) were intravenously injected into each tumor-bearing mouse. Then, the mice were sacrificed at 1, 24, and 48 h post-injection, respectively. The major organs (heart, liver, spleen, lung, and

kidney) and tumors were harvested, cut into small pieces, and digested with *aqua regia* for a week. The amount of Mo in these organs was analyzed by ICP-OES.

***In Vivo* Antitumor Therapeutic Efficacy:** When the tumors grew to approximately 50 mm^3 in volume, the mice were divided into seven groups randomly with 7 mice in each group as follows: Group 1, PBS (0.1 mL); Group 2, PPDA@MoS_2 ([Mo] = 1 mg/mL, in 0.1 mL PBS); Group 3, free 1-MT (1.12 mg/mL, in 0.1 mL phosphate buffer, pH 5.0); Group 4, free cisplatin (0.32 mg/mL, in 0.1 mL PBS); Group 5, PPDA@MoS_2 ([Mo] = 1 mg/mL, in 0.1 mL PBS) + laser; Group 6, Pt-PPDA@MoS_2 ([Mo] = 1 mg/mL, in 0.1 mL PBS) + laser; and Group 7, 1-MT-Pt-PPDA@MoS_2 ([Mo] = 1 mg/mL, in 0.1 mL PBS) + laser. All groups except Group 3 were treated through tail-vein intravenous injection, while Group 3 was treated through intratumoral injection. The injection dose was applicable for each mouse. The administered free cisplatin and 1-MT had equivalent doses of the respective drug-loaded complexes. For PPT, the tumor of each mouse was irradiated by an NIR laser (808 nm, 1 W/cm^2, and 5 min) at 2.5 h post-injection. We treated the mice on the first day, the fourth day and the eighth day. The tumor size and body weight were monitored every two days. The tumor volume (V, mm^3) was calculated using the formula of $V = 3\pi ab^2/4$, where a and b refer to the length and width of the tumor, respectively. Relative tumor volume was calculated as V/V_0 (V_0, tumor volume before treatment; V, tumor volume after treatment at a given time point). Tumors and major organs (heart, liver, spleen, lungs, and kidneys) were surgically harvested at the end of the treatment. The tissues were collected, fixed, sectioned, and hematoxylin & eosin (H&E) or terminal deoxynucleotidyl transferase-mediated dUTP-biotin nick end labeling (TUNEL) stained. The tumor tissues were also sectioned for CD4, CD8, and FOXP3 immunostaining, while the spleens were sectioned for CD4 and CD8 immunostaining. HMGB-1 and CRT in tumors were then analyzed by the immunohistochemistry according to the literature protocols.[4]

Cytokines Detection: Serum samples were isolated from mice on day 14 and diluted for analysis. Tumor necrosis factor α (TNF-α), interferon-γ (IFN-γ), interleukin 6 (IL-6), and Kynurenine (Kyn) were analyzed with ELISA kits according to the literature.[5]

***In Vivo* Tumoral Distribution of Immune Cells:** The mice were killed by cervical dislocation and immersed in 75% alcohol for 2-5 min. The tumors were obtained under sterile conditions, extracted, and stored in PBS solution. The tumor tissues were grinded in a 200-mesh sieve to obtain a single cell suspension of tumor-infiltrating tissue. Then the lymphocytes suspension in the tumor tissues was obtained according to the instructions of the Tumor Infiltrating Lymphocyte Cell Separation Medium Kit, Mice. The lymphocytes separation solution and an equal volume of prepared single cell suspension were mixed and then centrifuged (500 g, 30 min) at room temperature. The second lymphocyte layer was separated, washed with 10-mL cell-washing solution, and collected. Then, the cells were resuspended in 5 mL of PBS and centrifuged (250 g, 10 min) to collect the tumor-infiltrating lymphocytes. The obtained lymphocytes were filtered by the nylon wool column for 6-8 times to obtain T cells. Further, the collected cells were incubated with IgG isotype control, anti-CD3-FITC, or anti-CD4-PE/anti-CD8-FITC antibodies according to the standard protocols to determine the contents of $CD4^+$ and $CD8^+$ T cells in the tumors through flow cytometry.

Table S1. Zeta potentials and hydrodynamic diameters of MoS_2, PDA@MoS_2, PPDA@ MoS_2, 1-MT-Pt-PPDA@ MoS_2 particles.

Sample	Hydrodynamic Size (nm)	zeta-potential (mV)	Polydispersity Index (PDI)
MoS_2	623.6 ±2.5	-40.3 ±2.3	0.33 ±0.12
PDA@ MoS_2	471.0 ±4.5	-32.5 ±3.4	0.27 ±0.15
PPDA@ MoS_2	268.9 ±3.7	5.53 ±1.4	0.18 ±0.11
1-MT-Pt-PPDA@ MoS_2	351.5 ±4.8	8.46 ±2.8	0.34 ±0.16

Table S2. DL and EE of cisplatin and/or 1-MT in different complexes.

Sample	DL (cisplatin)	EE (cisplatin)	DL (1-MT)	EE (1-MT)
1-MT-PPDA@ MoS_2	/	/	60.3%	37.6%
Pt-PPDA@ MoS_2	33.2%	14.2%	/	/
1-MT-Pt-PPDA@ MoS_2	32.1%	9.3%	55.8%	32.5%

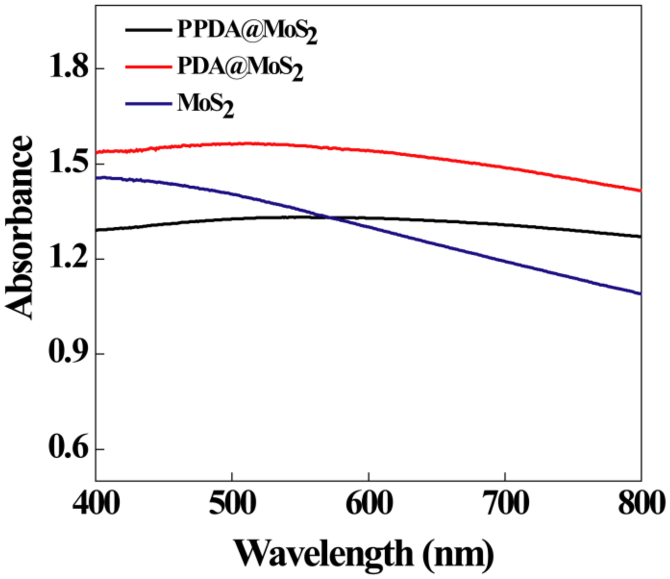

Figure S1. UV-vis spectra of MoS_2, PDA@MoS_2 and PPDA@MoS_2 particles.

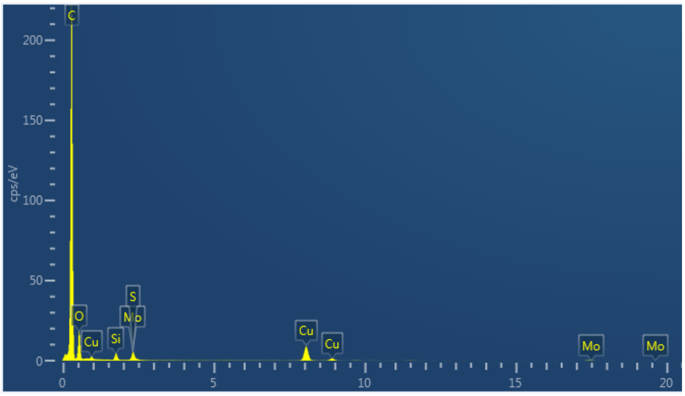

Figure S2. EDS spectrum of PDA@MoS_2 hybrids.

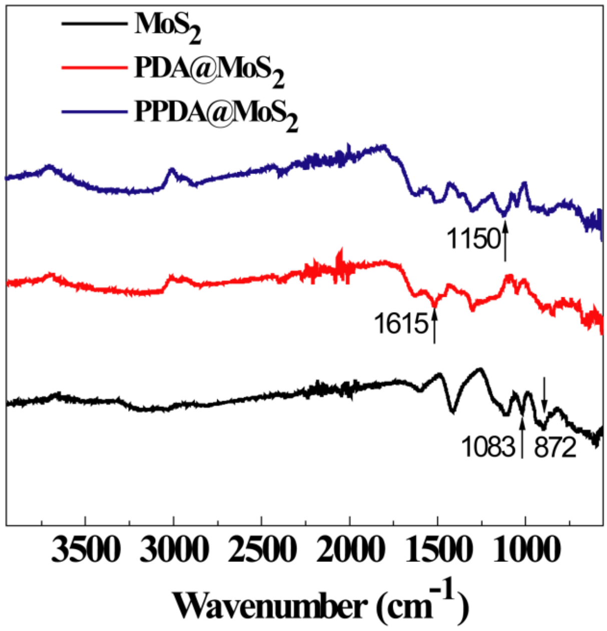

Figure S3. FTIR spectra of MoS_2, PDA@MoS_2 and PPDA@MoS_2 particles.

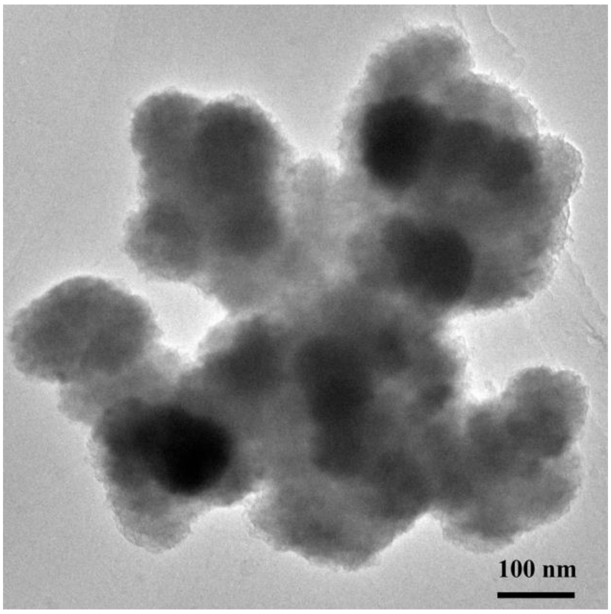

Figure S4. TEM image of 1-MT-Pt-PPDA@MoS_2 complexes.

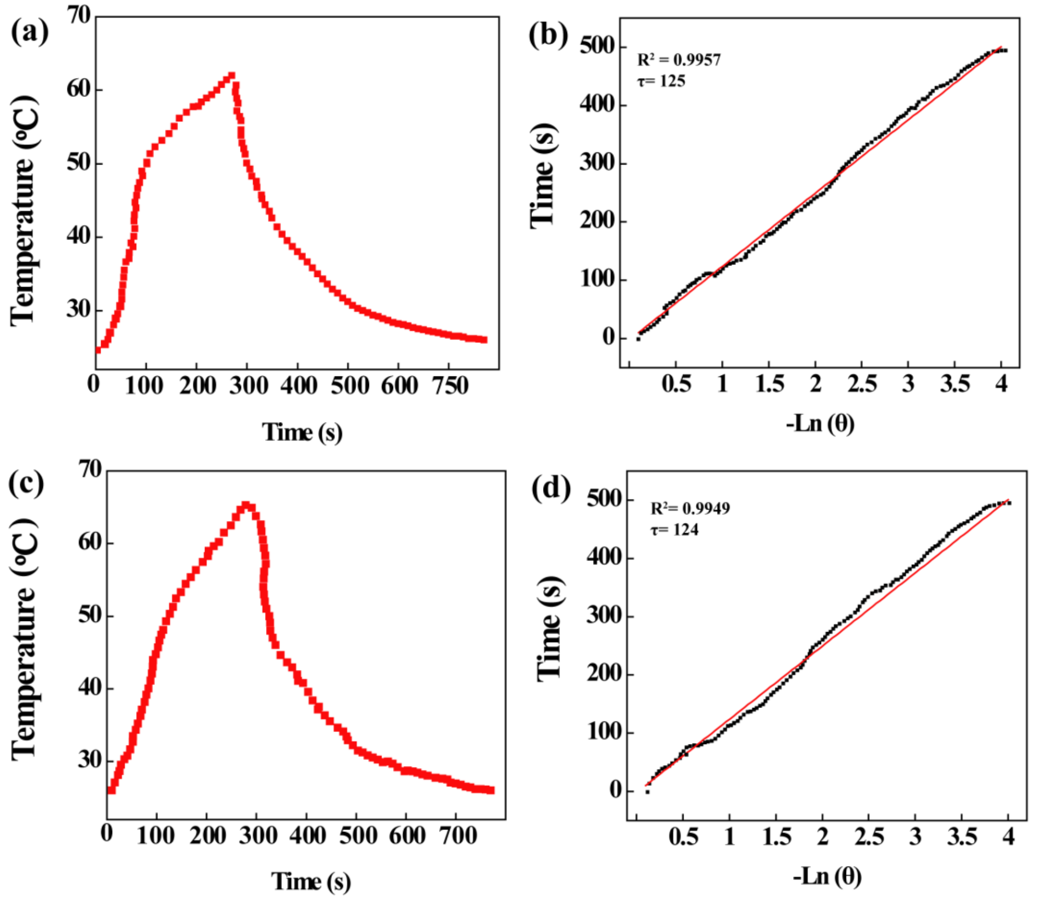

Figure S5. Temperature change of the 1-MT-Pt-PPDA@MoS_2 (a) and PPDA@ MoS_2 (c) aqueous solution irradiated by an 808-nm laser (1.0 W/cm^2) for 300 s. Plot of the cooling time vs – Ln (θ) of the 1-MT-Pt-PPDA@MoS_2 (b) and PPDA@MoS_2 (d).

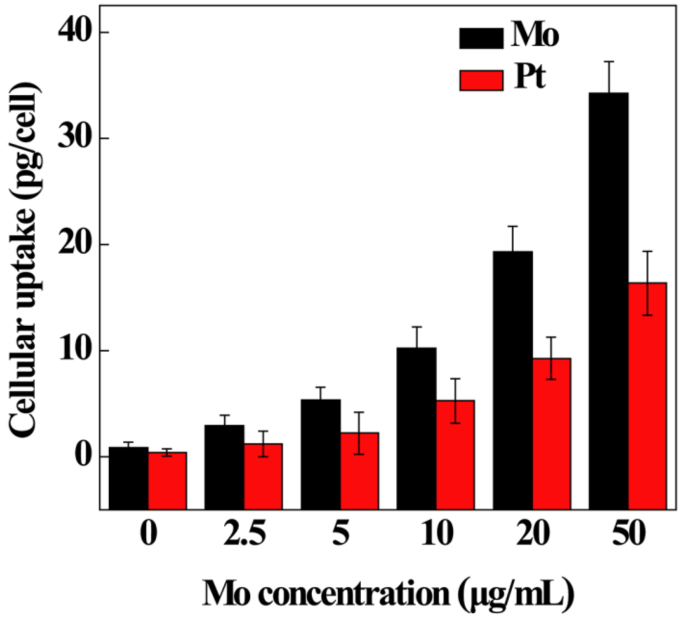

Figure S6. The Mo and Pt uptake by B16 cells after 6 h incubation of the 1-MT-Pt-PPDA@MoS_2 complexes at different Mo concentrations (n = 3).

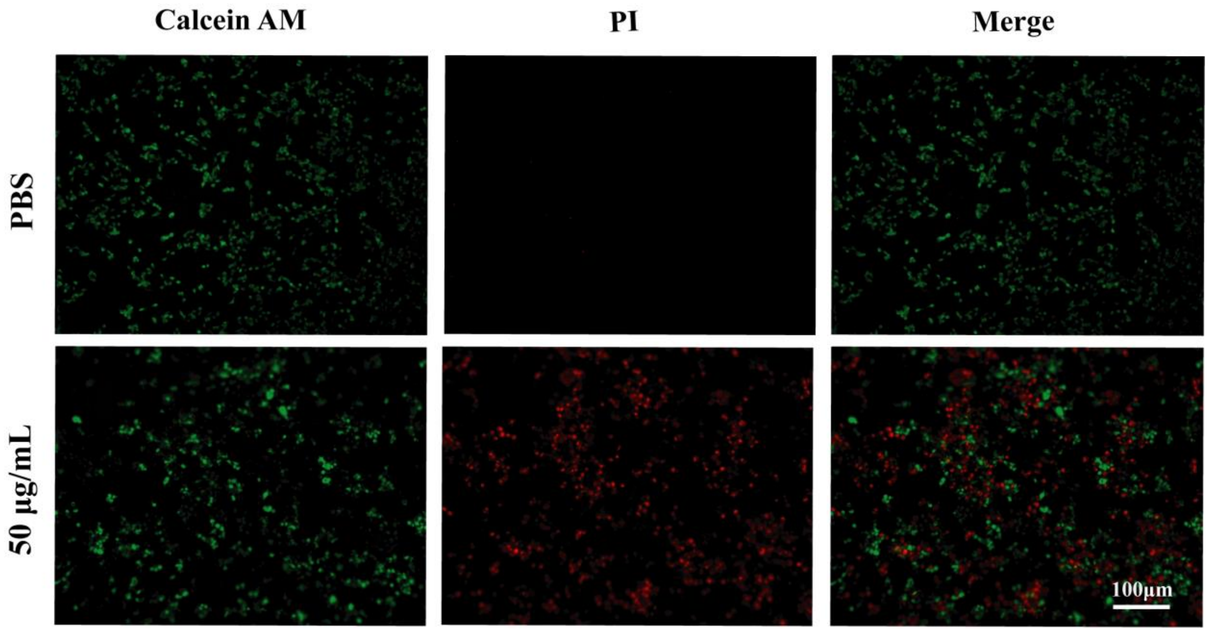

Figure S7. Fluorescence microscopic images of calcein AM and PI-costained B16 cells after treated with the 1-MT-PPDA@MoS2 complexes or PBS under the irradiation of an 808-nm laser (1 W/cm^2, 5 min). Scale bar represents 100 μm for each panel.

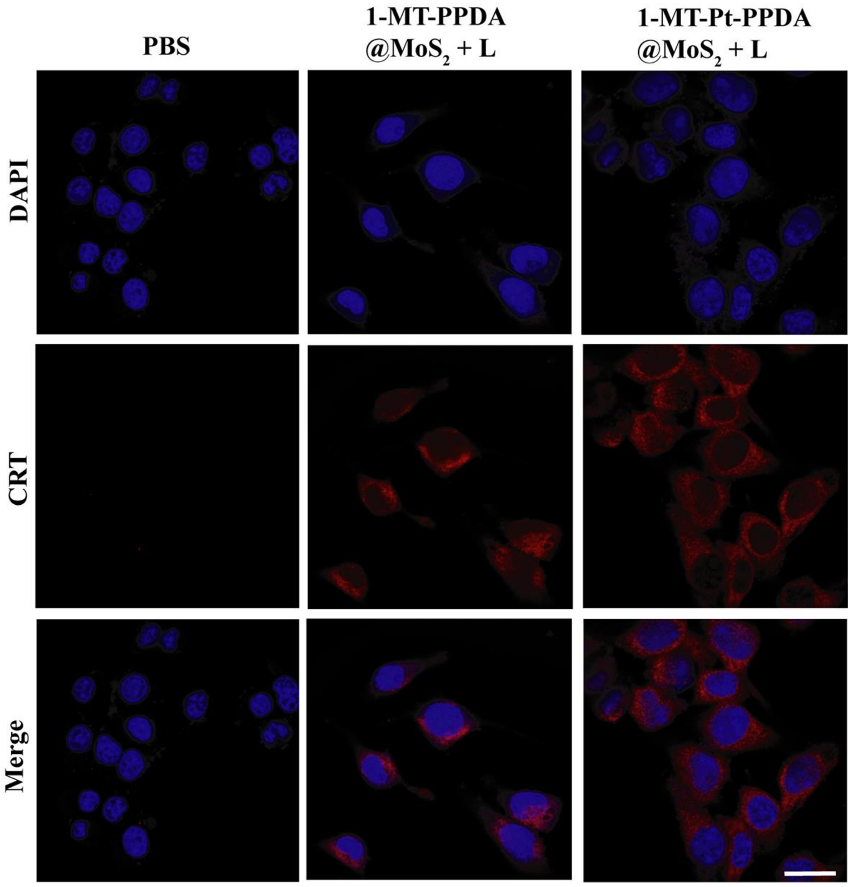

Figure S8. Immunofluorescence detection of CRT expressed on the surface of B16 cells as observed by confocal microscopy. Scale bar represents 20 μm for each panel.

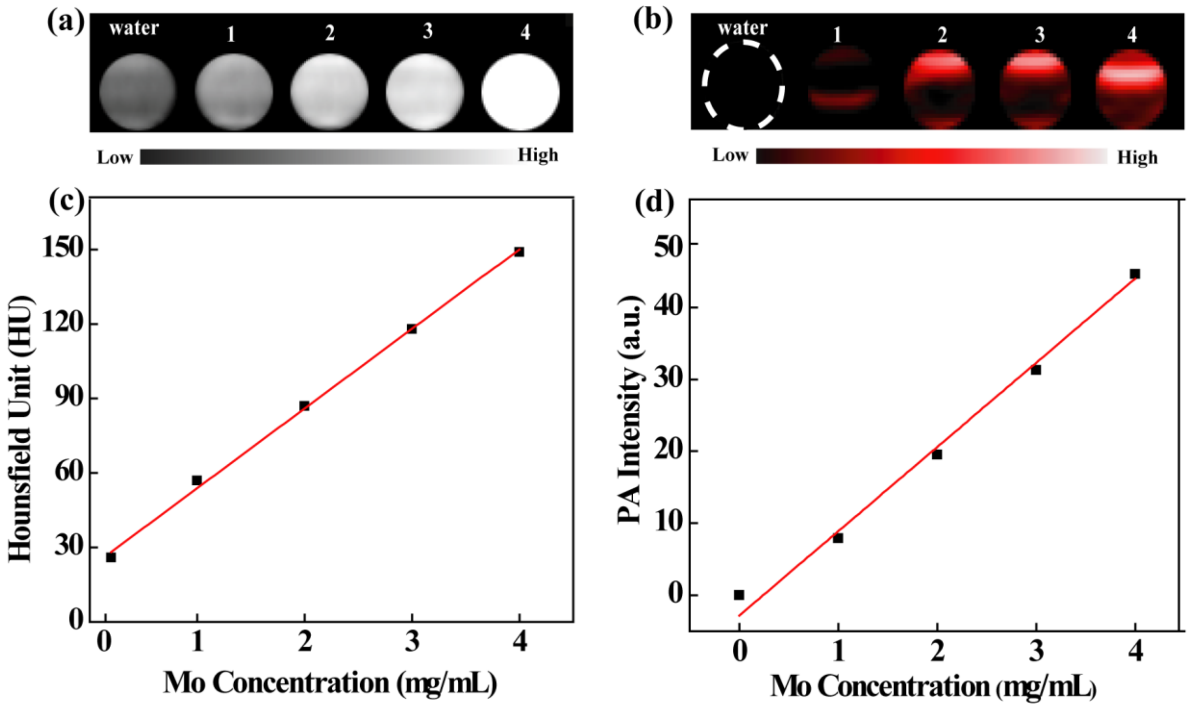

Figure S9. CT (a) and PA (b) images of 1-MT-Pt-PPDA@MoS2 and the corresponding plots of CT (c) and PA (d) value as a function of Mo concentration.

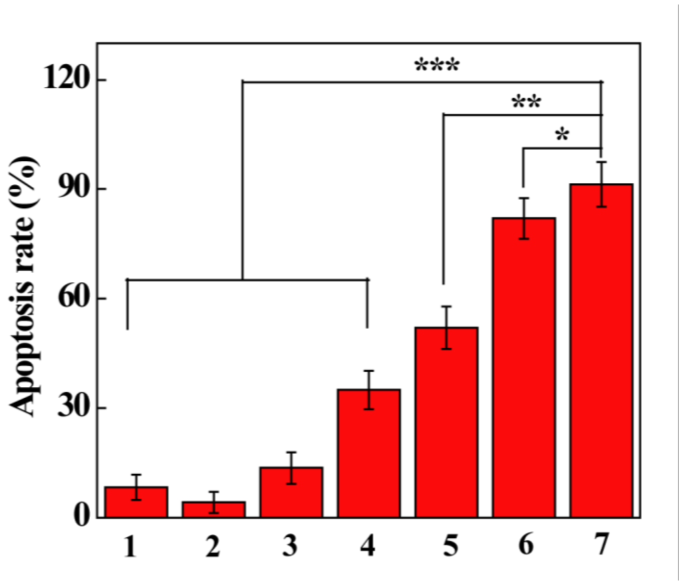

Figure S10. Apoptosis rate of B16 cells in tumors after different treatments. Group 1, PBS; Group 2, PPDA@MoS2; Group 3, free 1-MT; Group 4, free cisplatin; Group 5, PPDA@MoS2 + laser; Group 6, Pt-PPDA@MoS2 + laser; and Group 7, 1-MT-Pt-PPDA@MoS2 + laser (n =3).

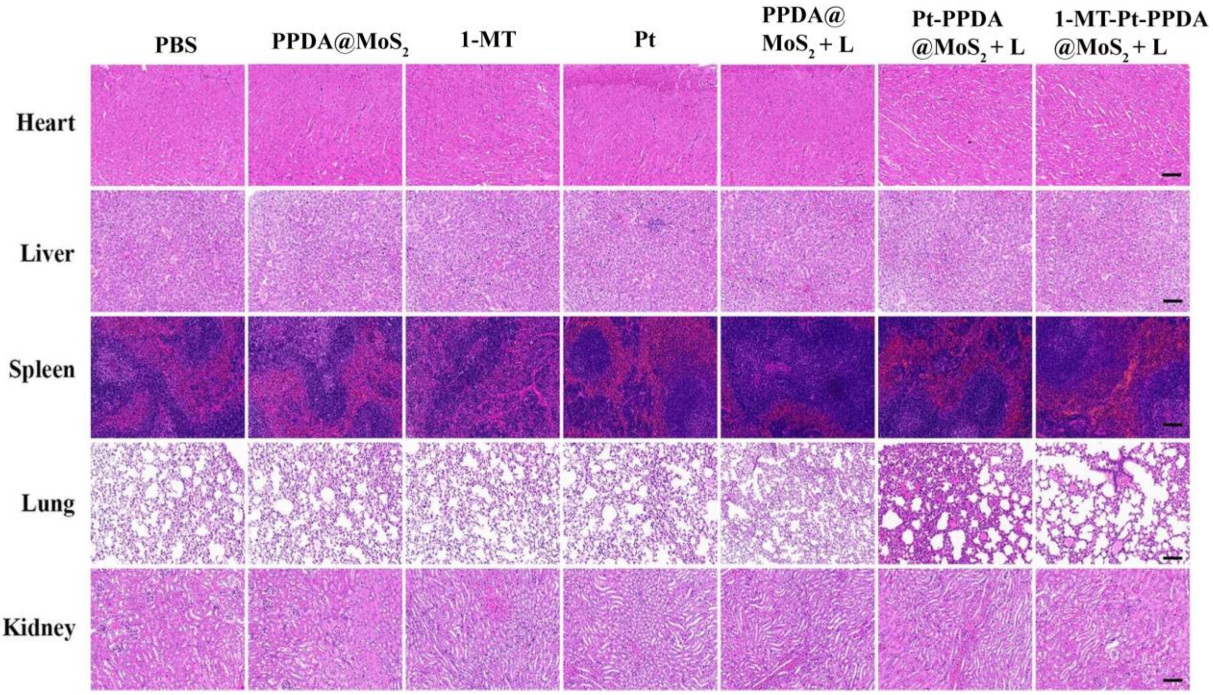

Figure S11. H&E staining of the sacrificed mouse organs after treatment with PBS, PPDA@MoS_2, free 1-MT, free cisplatin, PPDA@MoS_2 + laser, Pt-PPDA@MoS_2 + laser, or 1-MT-Pt-PPDA@MoS_2 + laser. Scale bar represents 100 μm for each panel.

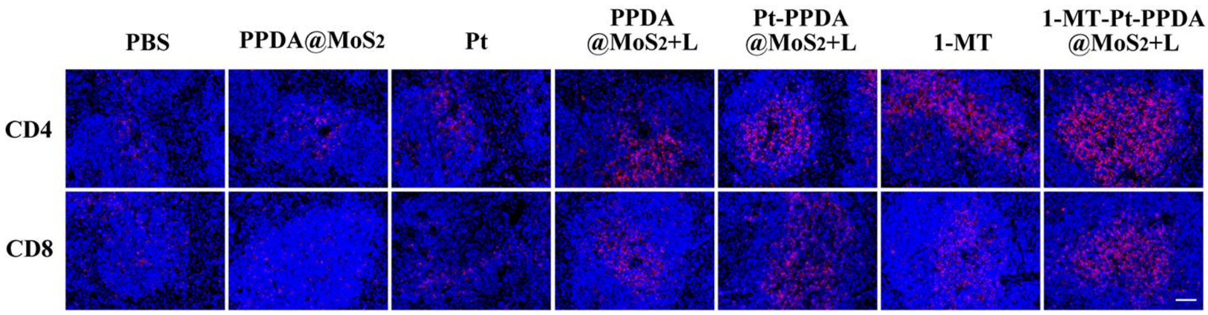

Figure S12. Representative immunofluorescence staining of CD4+ and CD8+ T cells in spleen. Scale bar represents 100 μm for each panel.

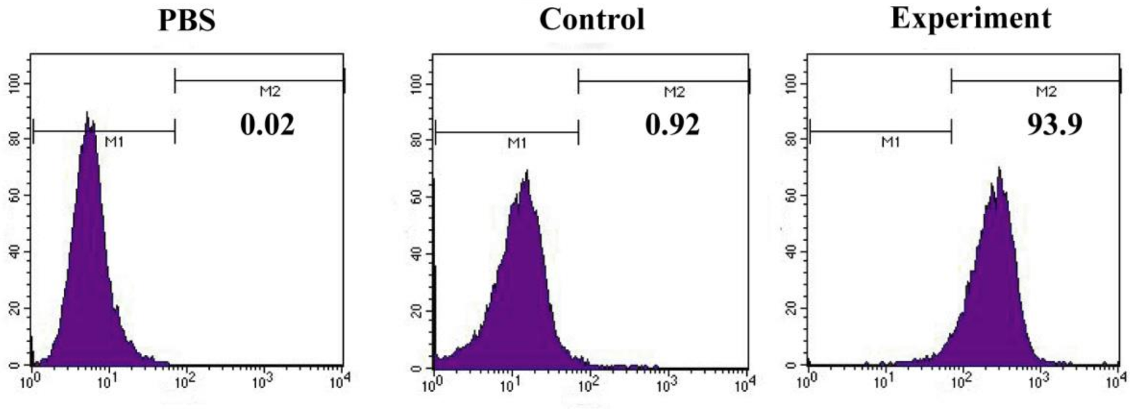

Figure S13. Flow cytometry detection of CD3 antibody-bound T cells in tumors.

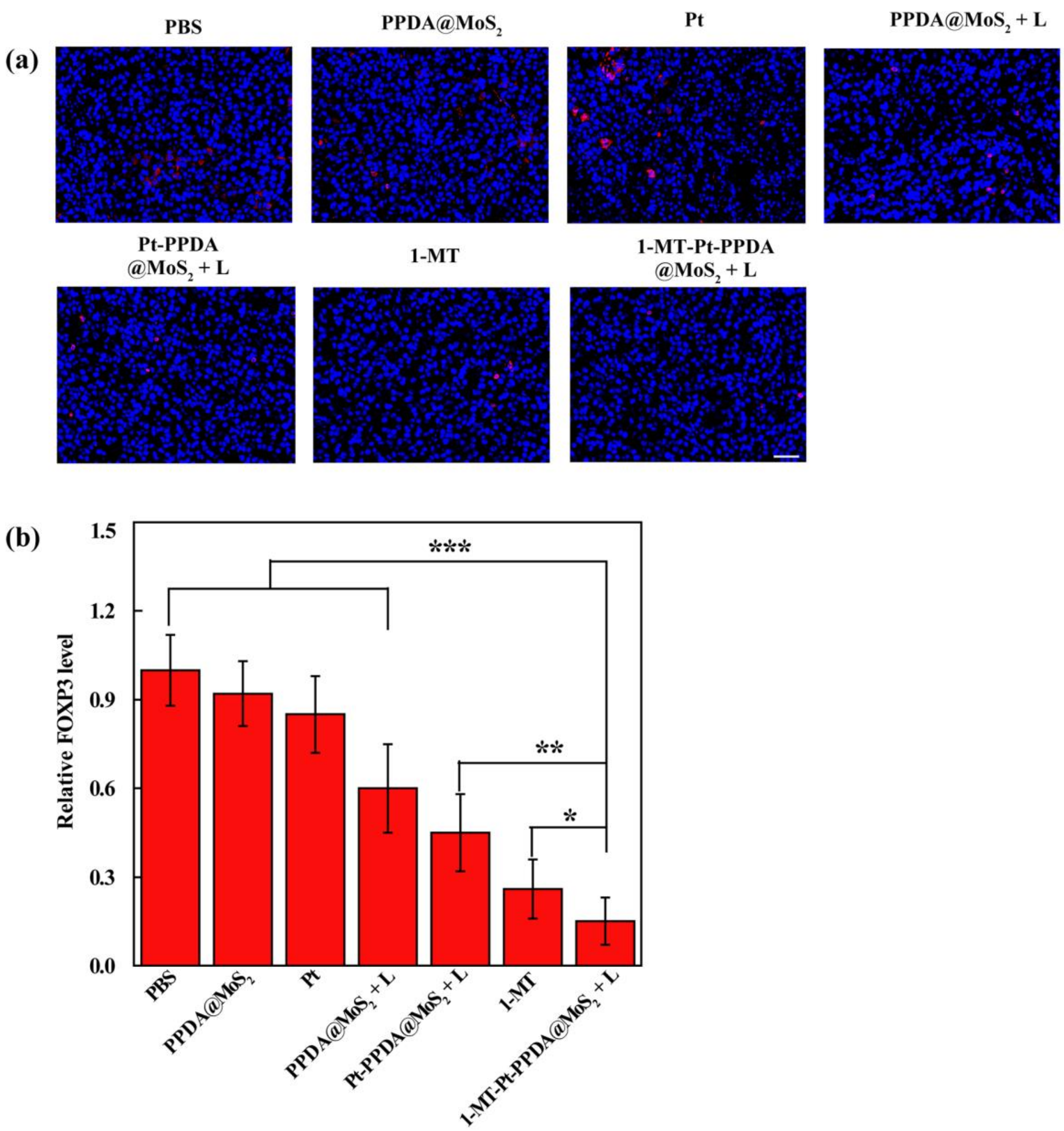

Figure S14. Immunofluorescence staining (a) and quantification analysis (b) of FOXP3+ T cells (Tregs) in tumors. Expression of FOXP3 was set to be 1.0 in cells treated with PBS (mean ± SD, n = 3). Scale bar represents 100 μm for each panel in (a).

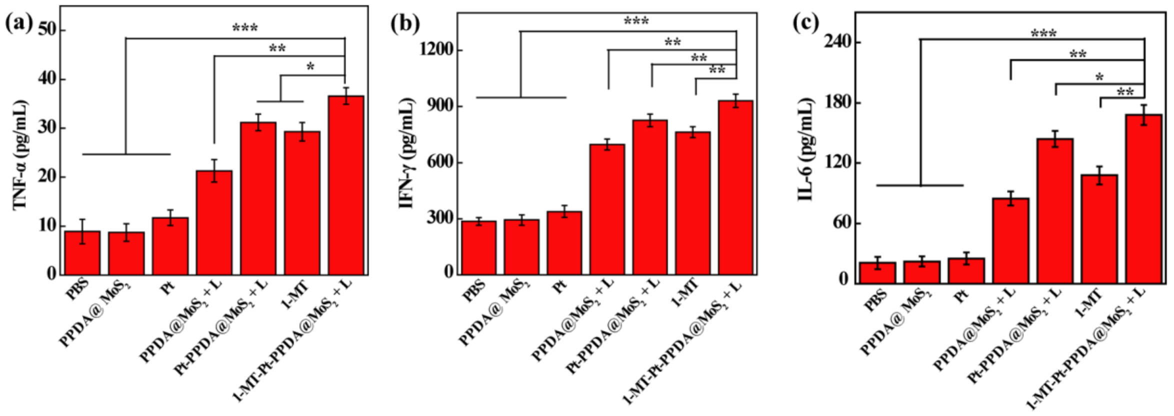

Figure S15. Contents of the TNF-α (a), IFN-γ (b) and IL-6 (c) in serum of mice on the 14th day after different treatments.

References

[1] L. D. Kong, L. X. Xing, B. Q. Zhou, L. F. Du, X. Y. Shi, *ACS Appl. Mater. Interfaces* **2017**, *9*, 15995.

[2] D. Li, Y. Fan, M. W. Shen, I. Banyai, X. Y. Shi, *J. Mater. Chem. B* **2019**, *7*, 277.

[3] Y. Fan, L. Z. Lin, F. F. Yin, Y. Zhu, M. W. Shen, H. Wang, L. F. Du, S. Mignani, J. P. Majoral, X. Y. Shi, *Nano Today* **2020**, *33*, 100899.

[4] Y. Tian, X. F. Wang, S. Zhao, X. Liao, M. R. Younis, S. J. Wang, C. N. Zhang, G. M. Lu, *ACS Appl. Mater. Interfaces* **2019**, *11*, 46626.

[5] C. Hu, T. Lei, Y. Z. Wang, J. Cao, X. T. Yang, L. Qin, R. Liu, Y. Zhou, F. Tong, C. S. Umeshappa, H. L. Gao, *Biomaterials* **2020**, *255*, 120159.
